# Reversible multicolor chromism in layered formamidinium metal halide perovskites

**DOI:** 10.1038/s41467-020-19009-z

**Published:** 2020-10-16

**Authors:** Bryan A. Rosales, Laura E. Mundt, Taylor G. Allen, David T. Moore, Kevin J. Prince, Colin A. Wolden, Garry Rumbles, Laura T. Schelhas, Lance M. Wheeler

**Affiliations:** 1 Center for Chemistry and Nanoscience, National Renewable Energy Laboratory, 15013 Denver West Parkway, Golden, CO 80401 USA; 2 SLAC National Accelerator Laboratory, 2575 Sand Hill Road, Menlo Park, CA 94025 USA; 3 Department of Chemical and Biological Engineering, Colorado School of Mines, Golden, CO 80401 USA; 4 Material Science Program, Colorado School of Mines, Golden, CO 80401 USA; 5 Renewable and Sustainable Energy Institute, Department of Chemistry, University of Colorado Boulder, Boulder, CO 80309 USA

**Keywords:** Electronic materials, Materials for energy and catalysis, Optical materials and structures

## Abstract

Metal halide perovskites feature crystalline-like electronic band structures and liquid-like physical properties. The crystal–liquid duality enables optoelectronic devices with unprecedented performance and a unique opportunity to chemically manipulate the structure with low energy input. In this work, we leverage the low formation energy of metal halide perovskites to demonstrate multicolor reversible chromism. We synthesized layered Ruddlesden-Popper FA_n+1_Pb_n_X_3n+1_ (FA = formamidinium, X = I, Br; *n* = number of layers = 1, 2, 3 … ∞) and reversibly tune the dimensionality (*n*) by modulating the strength and number of H-bonds in the system. H-bonding was controlled by exposure to solvent vapor (solvatochromism) or temperature change (thermochromism), which shuttles FAX salt pairs between the FA_n+1_Pb_n_X_3n+1_ domains and adjacent FAX “reservoir” domains. Unlike traditional chromic materials that only offer a single-color transition, FA_n+1_Pb_n_X_3n+1_ films reversibly switch between multiple colors including yellow, orange, red, brown, and white/colorless. Each colored phase exhibits distinct optoelectronic properties characteristic of 2D superlattice materials with tunable quantum well thickness.

## Abstract (web summary)

Metal halide perovskites feature crystalline-like electronic band structures and liquid-like physical properties that allow chemical manipulation of the structure with low energy input. Here, the authors leverage the low formation energy of 2D metal halide perovskites to demonstrate films that reversibly switch between multiple colors using tunable quantum well thickness.

## Introduction

Metal halide perovskite (MHP) materials have captured the imagination of the scientific community^1–5^. The coexistence of crystalline-like electronic band structure and liquid-like physical properties of MHP materials endows them with long carrier lifetimes, long carrier diffusion lengths, and exceptional defect tolerance to enable an array of exciting optoelectronic applications^6^. However, it is challenging to prevent changes in phase or composition of these “crystalline liquids^7^” even in benign conditions. Despite issues with stability upon exposure to humidity, heat, and light^8–10^, MHPs have enabled solar cells with a record power conversion efficiency (PCE) > 25%^11^.

Three-dimensional (3D) MHPs are composed of corner-sharing [MX_6_]^4−^ octahedra (M = Pb^2+^, Sn^2+^; X = I^−^, Br^−^, Cl^−^) that form an isotropic inorganic framework separated by A-site organic (methylammonium (MA), formamidinium (FA), etc.) or inorganic (alkali metal) monovalent cations. 2D MHPs also exhibit corner-sharing [MX_6_]^4−^ octahedra but only in two dimensions with the third dimension separated in space by the A-site cation^12–14^. 2D MHPs are classified as Ruddlesden-Popper^15^ or Dion-Jacobson^16,17^ phases of the general formula A′_2_A_n−1_M_n_X_3n+1_ or A′A_n−1_M_n_X_3n+1_, respectively, where A′ = 1+ or 2+ cation, A = 1+ cation, and *n* = 1, 2, 3, … ∞ is the number of connected 2D octahedra layers.

Chromogenic compounds change visible color when subjected to an external stimulus such as light, temperature, electric potential, solvent/vapor, or mechanical forces^18–20^. Materials that chromogenically change under these stimuli are classified as photochromic, thermochromic, electrochromic, solvatochromic, and mechanochromic, respectively^18^. Realizing reversible chromism in MHPs unlocks a new class of functional materials that couples a dynamic element to their remarkable optoelectronic properties. We envision dynamically tunable semiconductors to have applications that span switchable photovoltaics^21,22^ to energy storage^23^ and neuromorphic computing^24^. To date, there are two recognized mechanisms for reversible chromism in MHP materials: (1) crystal phase transformation^22^ and (2) molecular intercalation^21^. Both of these mechanisms have enabled the first examples of switchable photovoltaic windows^21,22^, which circumvent the fundamental trade-off between power conversion and visible light transmittance of traditional photovoltaic windows^25^.

MHPs are inherently thermochromic materials exhibiting significant optical changes induced by crystal phase transitions between the α-phase, a black high-symmetry perovskite phase composed of corner-sharing [MX_6_]^4−^ octahedra, and the δ-phase, a yellow-to-colorless non-perovskite hexagonal or orthorhombic phase composed of face- or edge-sharing [MX_6_]^4−^ octahedra, respectively^26–28^. Utilizing this phase transformation, the mixed-halide perovskite CsPbI_0.5_Br_2.5_ was employed in switchable photovoltiac window that achieved a 4.69% PCE with a phase transition temperature of 105 °C^22^. However, it is challenging to achieve transition temperatures below 100 °C for thermochromic window applications, and the smaller thermodynamic driving force at low temperature results in many hours for a complete phase transition^22^. In contrast, exposure of MHPs to vapor molecules that interact with the lattice can greatly decrease the temperature necessary for chromogenic behavior and improves transition speeds to seconds or minutes instead of hours as a result of the low formation/dissociation energy inherent in MHPs^29–31^. We have shown previously that exposure of MAPbI_3_ to methylamine vapor generates the clear MAPbI_3_•xCH_3_NH_2_ complex, which can be switched back to black MAPbI_3_ when heated with solar illumination^21^. Others have shown that room temperature solvatochromism is possible through the intercalation of ethanol into 2D OA_2_MAPb_2_I_7_^32^ or by forming reversible hydrates for the transition between 3D MAPbBr_3_ and 0D MA_4_PbBr_6_•2H_2_O^33^. FA-based MHPs, unlike MA analogues, do not exhibit hydrate phases. Instead, exposure of α-FAPbI_3_ to H_2_O catalytically converts the film into δ-FAPbI_3_ by interacting with the anisotropically strained (111) α-FAPbI_3_ lattice plane^34–36^.

In this work, we synthesize composite films composed of layered FA-based MHPs of the general formula FA_n+1_Pb_n_X_3n+1_ (X = I, Br) and their mixed-halide compositions. We leverage their liquid-like physical properties to demonstrate a third mechanism of dynamic chromism in MHPs through reversible layer formation and coalescence to form compounds that span 2D FA_2_PbX_4_ (*n* = 1) to 3D α-FAPbX_3_ (*n* = ∞) and finally 1D δ-FAPbI_3_. Reversible chromism is enabled by a second “reservoir” phase in the film composed of excess FAX salt, which allows FAX to reversibly shuttle between the reservoir and MHP layers. The mechanism is controlled by modulating the strength and number of H-bonds between the reservoir, MHP, and solvent vapor. Unlike previous reports on switchable MHPs that only switch between a single dark and a single light color^21,22^, our composite FA_n+1_Pb_n_X_3n+1_ films reversibly switch between a continuum of colors spanning yellow, orange, red, brown, and white/colorless. The colored phases are 2D superlattice materials with tunable quantum well thickness.

## Results

### Synthesis of layered formamidinium metal halide perovskites

Synthesis methods for free-standing powder and thin films of layered formamidinium metal halide perovskites were developed. We synthesized FA_n+1_Pb_n_X_3n+1_ powder by ball-milling a mixture of 4:1 FAX:PbX_2_ for 60 min in an inert atmosphere. Wide-angle X-ray scattering (WAXS) data shows that FA_n+1_Pb_n_I_3n+1_ powder exhibits Bragg diffraction peaks that correspond to a mixture of 2D FA_2_PbI_4_ (*n* = 1) with staggered octahedral layers and 3D α-FAPbI_3_ (3C/3R) that corresponds to *n* ≥ 2 (Supplementary Fig. 1). Though predicted to exist^37^, we believe this is the first report of iodide compositions of FA_n+1_Pb_n_X_3n+1_, and our WAXS results are in excellent agreement with previously reported MA_2_PbI_4_^38^ and FA_2_PbBr_4_^39^. Several different structures have been observed during the formation of FA-based halide perovskites, including 2H, 4H, 6H, 3C, and 3R structures (Supplementary Fig. 2)^40^. Our WAXS data eliminates any significant contributions from hexagonal structures (2H, 4H, 6H). In the FA_n+1_Pb_n_X_3n+1_ compounds observed here, FA^+^ molecules separate the 2D layers rather than the long-chain or bulky cations typically used in other Ruddlesden-Popper phases, such as butylammonium (13.4 Å), phenylethylammonium (16.6 Å), and hexylammonium (18.4 Å)^41,42^. Here we observe a smaller interlayer spacing of 8.9 Å, determined from the (001) peak of FA_2_PbI_4_, which is approximately the length of two FA^+^ molecules (8.2 Å, see Methods section).

**Fig. 1:**
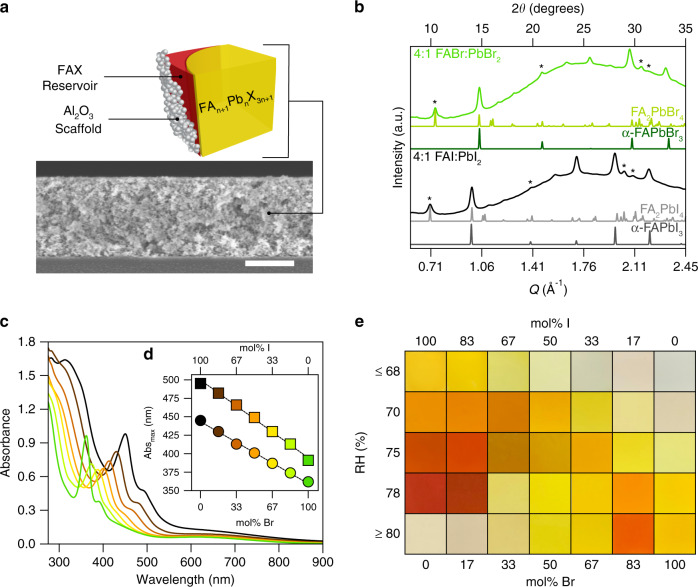
FA_n+1_Pb_n_X_3n+1_ composite film characterization and reversible chromism. **a** Illustration showing the components of a FA_n+1_Pb_n_X_3n+1_ composite film, including FA_n+1_Pb_n_X_3n+1_, FAX reservoir, and Al_2_O_3_ scaffold. Scale bar is 1 µm. **b** Wide angle X-ray scattering (WAXS) patterns of composite FA_n+1_Pb_n_X_3n+1_ films. The 2*θ* axis in (**b**) is relative to Cu Kα (1.5406 Å, 8.04 eV) radiation and was calculated from *Q* = 4*π*sin(*θ*)/*λ* where *λ* is the excitation wavelength. The background scattering in (**b**) is caused by the Al_2_O_3_ scaffold and glass substrate. * = FA_2_PbX_4_. Reference patterns of α-FAPbI_3_ were obtained from the Materials Design Group^78^, and the reference pattern of FA_2_PbI_4_ was obtained from ref. ^37^. Reference pattern of FA_2_PbBr_4_ was generated from FA_2_PbI_4_ by substituting Br in place of I and multiplying the unit cell volume by the Br/I radius ratio. **c** Absorbance spectra and **d** absorbance maxima as a function of composition for FA_n+1_Pb_n_X_3n+1_ films. Circles in **d** correspond to the strong excitonic peak tunable between 450 and 360 nm (*n* = 1) while squares correspond to the absorption edge tunable between 500 and 390 nm (*n* > 1), respectively. **e** Representative optical photographs of composite FA_n+1_Pb_n_X_3n+1_ films highlighting the diverse colors obtainable upon exposure to humidity. [PbI_2_] = 0.75 M in dimethyl sulfoxide (DMSO) was held constant in all precursors, which were spun onto a 1.58 ± 0.02 µm Al_2_O_3_ scaffold and annealed at 60 °C for 10 min. Note that films rich in iodide (>80% I) or bromide (>67% Br) bleach to white/transparent upon exposure to >80% or <68% relative humidity (RH), respectively.

We produce functional thin films and expand to bromide alloys by developing a scaffold composite composed of FA_n+1_Pb_n_X_3n+1_, an FAX reservoir, and a porous Al_2_O_3_ nanoparticle scaffold (Fig. 1a). Composite FA_n+1_Pb_n_X_3n+1_ films are synthesized by spin-coating a precursor solution containing 3 M FAX and 0.75 M PbX_2_ (4:1 FAX:PbX_2_; X = I, Br) in DMSO onto a 1.58 ± 0.02 μm-thick Al_2_O_3_ scaffold. FA_n+1_Pb_n_X_3n+1_ domains are formed by annealing the spin-coated film at 60 °C for 10 min. WAXS of composite FA_n+1_Pb_n_X_3n+1_ films (Fig. 1b) match FA_n+1_Pb_n_I_3n+1_ powder produced by ball-milling (Supplementary Fig. 1) and both exhibit a mixture of Bragg diffraction peaks that correspond to 2D FA_2_PbX_4_ (*n* = 1) with staggered octahedral layers and 3D α-FAPbX_3_ (3C/3R). Specifically, we observe Bragg diffraction peaks at 0.703 Å^−1^, 1.400 Å^−1^, 2.040 Å^−1^, and 2.095 Å^−1^ for 100% I and 0.738 Å^−1^, 1.478 Å^−1^, 2.157 Å^−1^, and 2.198 Å^−1^ for 100% Br that correspond to the (001), (002), (201), and (003) planes of 2D FA_2_PbX_4_ (*n* = 1) (Fig. 1b, asterisks). The 100% Br sample exhibits peaks shifted to higher *Q* due to the smaller lattice constant associated the bromide anion.

The optically active crystalline FA_n+1_Pb_n_X_3n+1_ (X = I, Br) domains are composed of predominantly *n* = 1 and *n* = 2 layers. FA_n+1_Pb_n_X_3n+1_ domains exhibit compositionally tunable optical properties that vary linearly as the halide ratio is varied from 100% iodide to 100% bromide (Fig. 1c, d) with two absorption peaks: (1) a strong excitonic peak tunable between 450 and 360 nm (circles) and (2) an absorption edge tunable between 500 and 390 nm (squares) that correspond to *n* = 1 and *n* = 2 layers, respectively. The optical properties observed for composite FA_n+1_Pb_n_X_3n+1_ films are consistent with other 2D MHPs^41,43–46^.

### Reversible chromism in layered FA-based MHPs

Composite FA_n+1_Pb_n_X_3n+1_ films show a brilliant array of reversible coloration upon exposure to solvent vapor. The visual appearance of composite FA_n+1_Pb_n_X_3n+1_ films range from colorless to yellow, orange, red, and brown (Fig. 1e). We attribute the mechanism of color change to a dynamic equilibrium between FAX salt pairs intercalated into the FA_n+1_Pb_n_X_3n+1_ domain or shuttled to an adjacent amorphous “reservoir” domain composed of excess FAX (Fig. 1e). The equilibrium is described by Eq. 1 (see Supplementary Note 1 for a derivation of *q*):

1 $${\mathrm{FA}}_{{\mathrm{n}} + 1}{\mathrm{Pb}}_{\mathrm{n}}{\mathrm{X}}_{3{\mathrm{n}} + 1} \leftrightarrow {\mathrm{FA}}_{{\mathrm{n}} + 1 - q}{\mathrm{Pb}}_{\mathrm{n}}{\mathrm{X}}_{{\mathrm{3}}n + 1 - q} + {\mathrm{qFAX}}$$

$$n = 1,2,3, \ldots \infty ;q = \frac{1}{{n(n + 1)}}$$

**Fig. 2:**
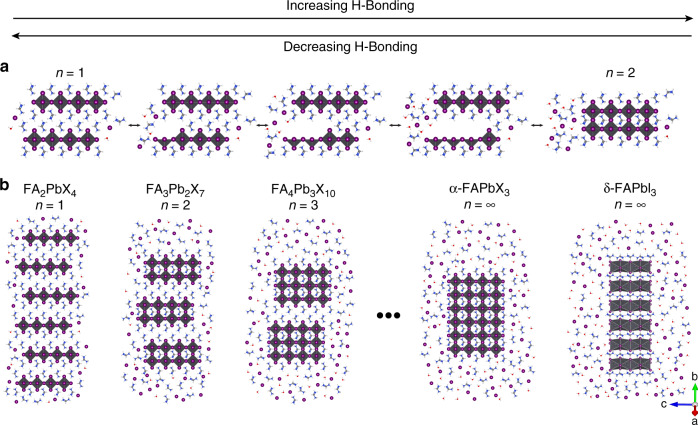
Mechanism of multicolor chromism in layered formamidinium metal halide perovskites. **a** Reversible FAX shuttling between the FAX reservoir and layered metal halide perovskite (MHP) causes layer formation and coalescence. **b** Illustration of the reversible switching mechanism in chromic composite FA_n+1_Pb_n_X_3n+1_ (X = I, Br) films enabled by a FAX reservoir adjacent to FA_n+1_Pb_n_X_3n+1_ domains. Each colored film is a mixture of multiple thicknesses (*n*).

The dynamic equilibrium is shifted by modulating the strength and number of H-bonds between solvent molecules, reservoir, and MHP. This insight allows us to design composite materials composed of FA_n+1_Pb_n_X_3n+1_ domains that change color in response to solvent vapor (solvatochromism) or temperature (thermochromism) by reversibly forming compounds that span 2D FA_2_PbX_4_ (*n* = 1) to 3D α-FAPbX_3_ (*n* = ∞) and finally 1D δ-FAPbI_3_ (Fig. 2).

There are three main drivers in the design of composite thin films: (1) FA_n+1_Pb_n_X_3n+1_ domains must be accessible to solvent vapor, (2) a reservoir phase must be present, and (3) the solvent vapor must interact favorably with FA_n+1_Pb_n_X_3n+1_. In order to provide solvent access, FA_n+1_Pb_n_X_3n+1_ domains are formed in a mesoporous Al_2_O_3_ nanoparticle scaffold. The scaffold limits the size of the FA_n+1_Pb_n_X_3n+1_ domains and provides a pathway for solvent vapor transport. The reservoir phase is formed by using an excess of FAX relative to PbX_2_ (>3:1 FAX: PbI_2_). Solvent molecules and water in the composite film are maintained by annealing at low temperatures (60 °C) in a 40% relative humidity (RH) atmosphere without exposure to dry-air flow. A detailed discussion and explanation for composite FA_n+1_Pb_n_X_3n+1_ film formation is supplied in Supplementary Note 2 and Supplementary Figs. 3–9. When assembled properly, composite films will reversibly change color rapidly (<1–20 min depending on solvent vapor flow rate) and over many cycles (>10). Though ball-milled powder samples show a chromogenic response to solvent vapor, the transitions are slower (30 min–1 h) with limited reversibility (Supplementary Fig. 10).

The final design criterium is choice of solvent vapor. Reversible chromism of composite FA_n+1_Pb_n_X_3n+1_ films is strongly dependent on the affinity of the solvent vapor to form H-bonds with the FAX reservoir and FA_n+1_Pb_n_X_3n+1_ constituents. A variety of solvent vapors were used to highlight the dependence on H-bonding (Supplementary Fig. 11). Chloroform and dichloromethane (DCM) have Lewis acidic protons that are H-bond donors but have no significant H-bond acceptor character. Tetrahydrofuran (THF) and pyridine have oxygen or nitrogen Lewis basic groups, respectively, that are H-bond acceptors. Composite FA_n+1_Pb_n_X_3n+1_ films exposed to chloroform, DCM, or THF do not noticeably change color, whereas pyridine bleaches the film to colorless. The stronger Lewis basicity of the pyridine compared to THF leads to solvation and complex formation with [PbX_6_]^4−^ to form isolated octahedra. The ability of the solvent to both donate and accept H-bonds is critical for reversible switching to occur. Solvent vapors such as water or alcohols (methanol, ethanol, and isopropyl alcohol) with H-bond-active hydroxyl groups will deeply color composite FA_n+1_Pb_n_X_3n+1_ films from yellow to orange, red, and brown (Supplementary Fig. 11).

### Molecular interactions in reversible chromism

H-bonds are the thermodynamic driving force for the reversible chromism in composite FA_n+1_Pb_n_I_3n+1_ films. Chromism is dependent on both the strength and number of H-bonds. For instance, color change induced by exposure to solvent vapor (solvatochromism) may be reversed by increasing the temperature of the film (thermochromism) if the vapor concentration is held constant. The thermochromic effect is a function of the strength of H-bonding in the system, as the relative strength of H-bonds is weakened when heated and strengthened when cooled. We observe this effect with only mild conditions where a bleached film will rapidly (<10 s) convert to brown upon heating the film to 35 °C (Supplementary Fig. 12), which nicely resides in the desirable range for thermochromic window applications^21^.

The remainder of the discussion centers around chromism induced by a change in the number of H-bonds, rather than strength, to manipulate the thermodynamic equilibrium of Eq. 1. We control the number of H-bonds by either changing the relative amount of the FA_n+1_Pb_n_X_3n+1_ phase to the reservoir phase or by changing the concentration of solvent vapor in the environment. We chose FA_n+1_Pb_n_I_3n+1_/water vapor as a model system since water vapor affords facile control over RH (concentration) compared to the partial pressure of other solvents and fast solvatochromic (hygrochromic) switching of <1–20 min depending on water vapor flow rate. A switching time of <7 s is shown in Supplementary Movie 1. We believe these observations are representative of the underlying mechanism, regardless of choice of chemical system.

**Fig. 3:**
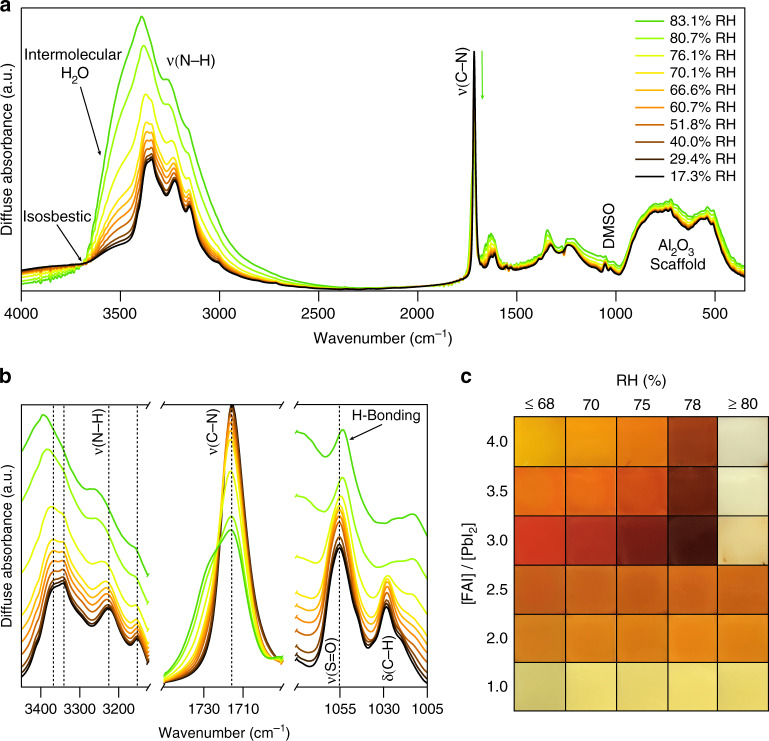
Molecular interactions are the thermodynamic driving force for reversible chromism. **a** Representative diffuse-reflectance infrared Fourier transform spectroscopy (DRIFTS) spectra collected at various relative humidity (RH) and extracted from Supplementary Fig. 13. **b** Spectra focused on the H-bonding regions of the composite film: N–H stretch region of FA^+^, C–N asymmetric stretch region of FA^+^, and the S=O stretch and C–H rocking regions of dimethyl sulfoxide (DMSO). Dashed vertical lines indicate peak location at 17.3% RH. **c** Optical photographs showing the effect of changing the number of molecular interactions on the hygrochromic properties by varying the FAI:PbI_2_ ratio and RH. [PbI_2_] = 0.75 M in DMSO was held constant in all precursors, which were spun onto a 1.58 ± 0.02 µm Al_2_O_3_ scaffold and annealed at 60 °C for 10 min. DRIFTS spectra and optical photographs were collected on films with a FAI:PbI_2_ ratio of 4.0.

We probe molecular interactions in the FA_n+1_Pb_n_I_3n+1_/water vapor model system by varying the RH between 17 and 83% RH in conjunction with in-situ diffuse-reflectance infrared Fourier transform spectroscopy (DRIFTS). The DRIFTS spectrum collected at 17.3% RH clearly shows vibrational modes corresponding to the components of the reservoir. The FAI is represented by N–H stretching modes between 3400 and 3200 cm^−1^ and C–N asymmetric stretching at 1716 cm^−1^ correspond to the FA^+^ cation^47^. We also observe DMSO, H_2_O, and the Al_2_O_3_ scaffold (Fig. 3a, black spectrum): C–H bending modes between 1400 and 1300 cm^−1^, S=O stretching at 1056 cm^−1^, and C–H rocking at 1028 cm^−1^ corresponding to DMSO^48^; H–O–H bending at 1630 cm^−1^ and O–H stretching at 3510 cm^−1^ corresponding to intermolecularly bound H_2_O^49,50^; and Al-O stretching between 1000 and 500 cm^−1^ corresponding to the Al_2_O_3_ scaffold^51^.

We increase the number of H-bonds by increasing RH from 17.3% to 83.1%. The film changes color from yellow to orange to brown to white/colorless (Fig. 3c, top row), and the DRIFTS spectra correspondingly undergo several changes. Before a change in color occurs (RH < 70%), the increased intensity of the O–H stretching mode located between 3550 and 3200 cm^−1^ suggests increasing amounts of H_2_O are incorporated into the film and are intermolecularly H-bonded with other H_2_O molecules or with FAI in the reservoir^49,50^. The isosbestic point at 3688 cm^−1^ signifies a reduction in scaffold-bonded H_2_O (>3688 cm^−1^) and an increase in intermolecularly H-bonded H_2_O or hydrated FAI (3550–3200 cm^−1^, Fig. 3a)^48^.

We observe a threshold value for chromism at c.a. 70% RH, which results in more dramatic changes to the DRIFTS spectra. The H-bond interactions shift the thermodynamic equilibrium (Eq. 1) from the FA_n+1_Pb_n_I_3n+1_ domains to the FAI reservoir, indicated by an increase in spectral intensity, peak shifting, and peak broadening at RH > 70%. The N–H stretching modes broaden and blueshift, indicating the bonding environment of FA^+^ is changing over time as it is shuttled from the FA_n+1_Pb_n_X_3n+1_ domain to the reservoir (Fig. 3b). The broadening is accompanied by a shoulder that grows in at 1730 cm^−1^ (Fig. 3b). The blueshift at high RH indicates both shortening and strengthening of the N–H bond^52^ as a result of H-bond formation. Both S=O stretch and C–H rocking modes of DMSO remaining in the film redshift at higher RH values, which is characteristic with H-bonding to water and a distinct change in bonding environment (Fig. 3b)^48^. Although solvatochromism in our ball-milled powder synthesized without solvent (Supplementary Fig. 10) shows that DMSO is not crucial to the reversible switching mechanism, DMSO may facilitate switching by providing H-bond acceptor sites. The molecular interactions observed in DRIFTS during color change from yellow to orange, brown, and colorless are reversed upon removal of water vapor (Supplementary Fig. 13). Removal of H-bond interactions shift the thermodynamic equilibrium (Eq. 1) from the reservoir back to the FA_n+1_Pb_n_I_3n+1_ domains.

The reservoir volume also dictates the number of molecular interactions and the resulting color of the FA_n+1_Pb_n_I_3n+1_ composite. Hygrochromic properties are only observed when the FAI:PbI_2_ ratio is >3.0, which suggests that enough FAI must be present to form both the reservoir and the layered FA_n+1_Pb_n_I_3n+1_. As the FAI:PbI_2_ ratio increases, the number of molecular interaction sites increases in the film. The color of the film changes from red to orange to yellow as the equilibrium is pushed to thinner layers (smaller *n*) with higher FAI:PbI_2_ ratios. Thus, by controlling the number of molecular interactions through varying both the RH and the FAI:PbI_2_ ratio, the chromic response of the film can be extensively controlled with a wide variety of colors obtainable, including yellow, orange, red, brown, dark brown, and white/transparent. We note the 2D nature of our films lead to significantly improved moisture stability as evidenced by repeated and reversible color cycling between 20 and 82% RH, storage at ≤40% RH in air for months, and storage at 75% RH in air for over one month.

### Structural evolution due to molecular interactions

**Fig. 4:**
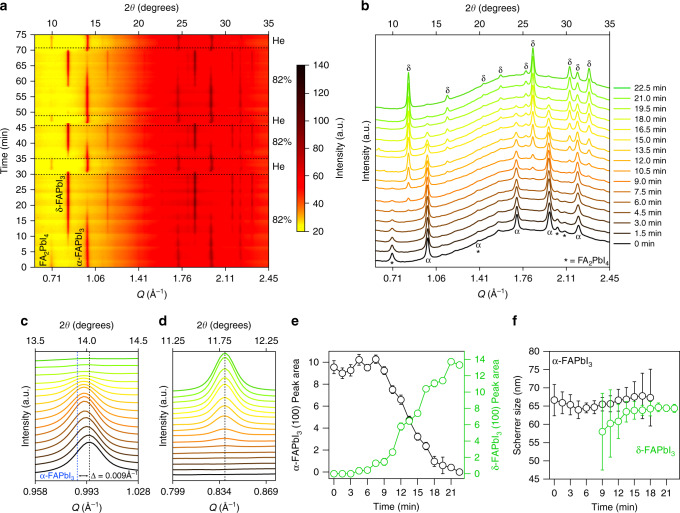
Structural evolution during reversible hygrochromic cycling. **a** In-situ wide-angle X-ray scattering (WAXS) data collected on a FA_n+1_Pb_n_I_3n+1_ composite film over three cycles of alternating exposure to 82% relative humidity (RH) and He flow. **b** Select WAXS data extracted from (**a**). Zoom-in on the region corresponding to the (100) peak of cubic α-FAPbI_3_ (**c**) and to the (100) peak of hexagonal δ-FAPbI_3_ (**d**). The 2*θ* axes are relative to Cu Kα (1.5406 Å, 8.04 eV) radiation and was calculated from *Q* = 4*π*sin(*θ*)/*λ* where *λ* is the excitation wavelength. The WAXS background scattering is caused by the Al_2_O_3_ scaffold and glass substrate, which is necessary for chromic properties as discussed in Supplementary Note 2. Comparison of cubic α-FAPbI_3_ (100) and hexagonal δ-FAPbI_3_ (100) peak area (**e**) and Scherrer crystallite size (**f**) as a function of time exposed to 82% RH. Fits and s.d. error bars in (**e**, **f**) were obtained by fitting the cubic α-FAPbI_3_ or hexagonal δ-FAPbI_3_ (100) peaks in (**b**) to a Voigt function.

We probe the structural response of FA_n+1_Pb_n_I_3n+1_ composites induced by molecular interactions using in-situ WAXS experiments. In-situ WAXS experiments nicely illustrate the reversible transformation from predominantly FA_n+1_Pb_n_I_3n+1_ domains with *n* = 1, 2 to α-FAPbI_3_ (*n* = ∞) and finally δ-FAPbI_3_. Starting at ambient RH, the films are exposed to humid air controlled at 82% RH. Dry conditions (~40% RH) are achieved by flowing Helium. The films are cycled through humid/dry conditions three times during collection of in-situ WAXS data (Fig. 4a). Flowing humid air at 82% RH causes Bragg peaks corresponding to FA_2_PbI_4_ and α-FAPbI_3_ to gradually disappear over 10.5 min and 18 min, respectively, while Bragg peaks corresponding to δ-FAPbI_3_ begin to emerge at 9 min. Disappearance of FA_2_PbI_4_ peaks in the WAXS pattern before α-FAPbI_3_ peaks disappear suggests *n* = 1 layers coalesce to form layers of *n* > 1.

Surface energy and strain are known to stabilize small or 2D grains of α-FAPbI_3_, whereas bulk crystals thermodynamically favor the non-perovskite δ-FAPbI_3_ hexagonal phases^34,53^. The strain of the domains is reduced as the domain grows and eventually transforms into the thermodynamically favored δ-FAPbI_3_ phase. We observe a strain-induced shift in the (100) peak of α-FAPbI_3_ from 0.995 Å^−1^ to 0.991 Å^−1^ (Δ = 0.004 Å^−1^) as RH is increased due to a relaxation of the growing α-FAPbI_3_ crystal between FAI layers (Fig. 4c)^34^. In contrast to the (100) peak of α-FAPbI_3_ at 0.995 Å^−1^, the (100) peak of δ-FAPbI_3_ at 0.838 Å^−1^ does not exhibit a shift in *Q* due to the relatively nonexistent strain in the 1D δ-FAPbI_3_ lattice (Fig. 4d)^34^. We note that the δ-FAPbBr_3_ phase is not observed at high RH (Supplementary Fig. 14) because δ-FAPbBr_3_ is only stable below −8 °C (265 K)^54^.

The WAXS data clearly shows hygrochromism in composite FA_n+1_Pb_n_I_3n+1_ films is not due to recrystallization of the material (dissolution of one phase followed by precipitation of another), but rather by reversible FAI shuttling between grains that maintain their initial size. We tracked the evolution of the (100) peak area for each species as a function of time exposed 82% RH (Fig. 4e) and quantified the size of the FA_n+1_Pb_n_I_3n+1_ domains using Scherrer analysis (Fig. 4f)^55^. The α-FAPbI_3_ (100) peak area decreases while the δ-FAPbI_3_ (100) peak area increases with an intersection occurring between 12-15 min, indicating the transformation of a single grain rather than the formation of a new one. 2D WAXS data collected during phase evolution shows the grains are polycrystalline and do not become textured over time (Supplementary Fig. 15), and the size of the domains remain constant as the FA_n+1_Pb_n_I_3n+1_ domains evolve with increasing RH (Fig. 4f). After exposure to flowing humid air at 82% RH until the film is white/colorless, exposure of flowing dry He reverses the observed phase transformations: the (100) peak of δ-FAPbI_3_ at 0.838 Å^−1^ disappears while the (100) peak of α-FAPbI_3_ at 0.995 Å^−1^, and the (001), (002), (201), and (003) peaks of FA_2_PbI_4_ at 0.703 Å^−1^, 1.400 Å^−1^, 2.040 Å^−1^, and 2.095 Å^−1^ reemerge (Fig. 4a). As we remove H_2_O from the film, FAI is re-inserted into the α/δ-FAPbI_3_ domains to re-form the layered FA_n+1_Pb_n_I_3n+1_ structure.

### Dynamic control of optoelectronic properties

**Fig. 5:**
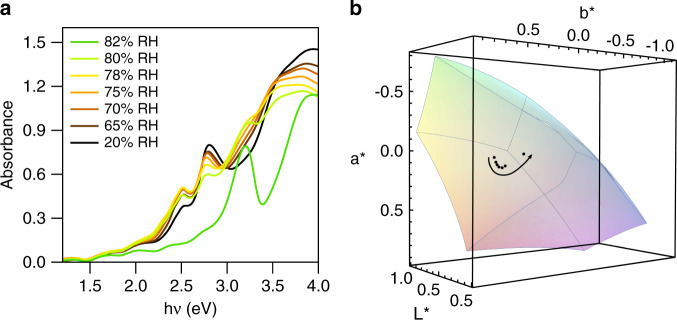
Color of hygrochromic films. **a** Absorbance and **b** Color of FA_n+1_Pb_n_I_3n+1_ composite films plotted in CIE 1976 L*a*b* coordinate space. Film color is calculated from absorbance spectra in (**a**). The colored polyhedron in **b** is a visualization of the sRGB gamut used in displays and digital photography. The arrow in **b** shows the direction of increasing relative humidity (RH).

Optical spectroscopy is used to probe the optical properties of the FA_n+1_Pb_n_I_3n+1_ domains as the *n* = 1 material coalesces to higher dimensional structures (*n* > 1). Our optical absorption and photoluminescence (PL) data confirm each observed color is a FA_n+1_Pb_n_I_3n+1_ mixture with multiple thicknesses that span *n* = 1 to *n* = ∞ (Fig. 5a). Varying the RH changes the relative ratios of n as the FA_n+1_Pb_n_I_3n+1_ domains trend toward thicker layers with increasing RH. Increasing the RH up to 78% results in a decrease of the excitonic peak at 2.79 eV and an increase in the absorption edge at 2.51 eV as well as the emergence of new absorption edges located at 2.30 eV, 2.07 eV, and 1.77 eV (Fig. 5a). Subjecting the film to 80% RH causes a decrease in all peaks as the film transforms to white/colorless. A dramatic absorption change is observed upon reaching 82% RH in which absorbance in the visible region is greatly reduced and a strong absorbance peak located at 3.18 eV emerges that is consistent with formation of hexagonal δ-FAPbI_3_^28,56^. This large distribution of absorption properties obtainable by varying RH is reflected in the CIE 1976 L*a*b* coordinate space that is representative of the human perception of color (Fig. 5b).

**Fig. 6:**
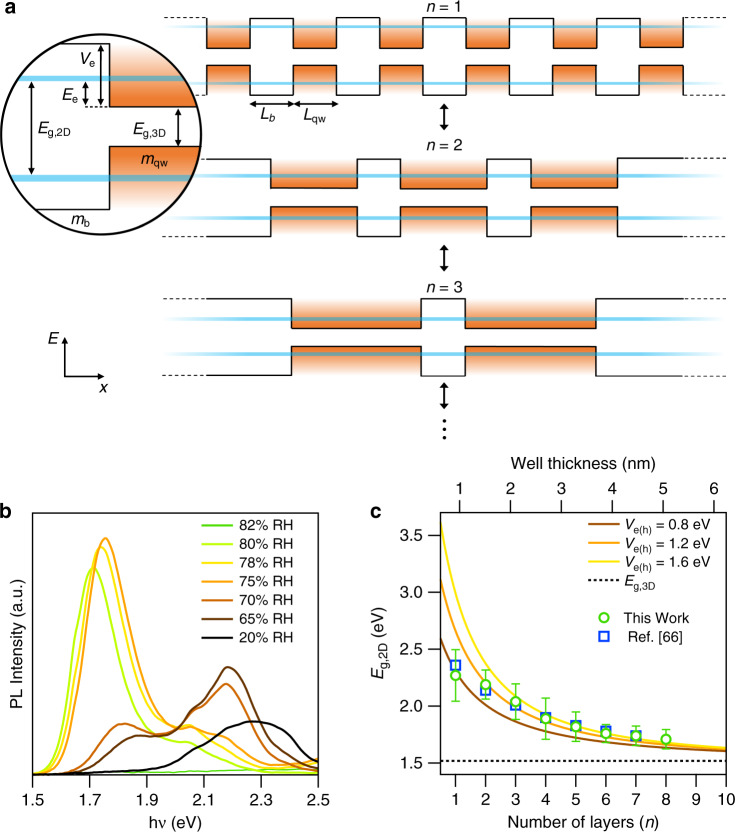
Superlattice description of FA_n+1_Pb_n_I_3n+1_ optical properties. **a** Diagram of the Kronig–Penney-like model used to describe FA_n+1_Pb_n_I_3n+1_ optical properties. **b** Photoluminescence (PL) of hygrochromic FA_n+1_Pb_n_I_3n+1_ films collected at various relative humidity (RH). Each spectrum exhibits multiple peaks due to a mixture of ‘n’ layers. **c** A plot of the peak PL position and models used to describe our PL data. Marker position corresponds to the peak of the PL spectra in (**b**), and the error bars are the full-width at half maximum of the peak. Model parameters are described in the text.

Discrete optical transitions observed in the absorbance spectra occur due to the separation or coalescence of 2D octahedra layers. The optical bandgap of 2D FA_n+1_Pb_n_I_3n+1_ materials increases monotonically as *n* approaches 1 due to formation of minibands in the quantum well superlattice structure that emerges from alternating layers of formamidinium and connected lead halide octahedra layers (Fig. 6a). The optical bandgap increase relative to 3D α-FAPbI_3_ is written as:

2 $$E_{{\mathrm{g}},2{\mathrm{D}}} = E_{{\mathrm{g}},3{\mathrm{D}}} + E_{\mathrm{e}} + E_{\mathrm{h}}$$

where $E_{{\mathrm{g}},3{\mathrm{D}}} = 1.52\,{\mathrm{eV}}$ is the bulk bandgap of α-FAPbI_3_^57^ and $E_{{\mathrm{e}}({\mathrm{h}})}$ is the minimum energy of the lowest-energy miniband. We determine $E_{{\mathrm{e}}({\mathrm{h}})}$ by adapting the Kronig–Penney (KP) model^58^ for an electron (hole) in a one-dimensional periodic potential. The KP-like model has successfully described conventional III–V superlattice structures^59,60^ and has recently been applied to MHP materials^61–63^. The dispersion relation for electrons (holes) in the *x* direction is:

3 $$\cos \left( {\beta L_{{\mathrm{qw}}}} \right)\cosh \left( {\alpha L_{\mathrm{b}}} \right) + \frac{1}{2}\left( {\gamma - \gamma ^{ - 1}} \right)\sin \left( {\beta L_{{\mathrm{qw}}}} \right)\sinh \left( {\alpha L_{\mathrm{b}}} \right) = {\mathrm{cos}}({\mathbf{k}}(L_{{\mathrm{qw}}} + L_{\mathrm{b}}))$$

where $L_{{\mathrm{qw}}}$ is the width of the metal halide quantum well layer, and $L_{\mathrm{b}}$ is the width of the barrier layer composed of formamidinium. Both widths are determined from XRD studies ($L_{{\mathrm{qw}}}$ = 0.624 nm and $L_{\mathrm{b}}$ = 0.690 nm). **k** is the superlattice wavevector, which is bound by $- \pi (L_{{\mathrm{qw}}} + L_{\mathrm{b}})$ and $\pi (L_{{\mathrm{qw}}} + L_{\mathrm{b}})$. The minimum energy of the lowest-energy miniband occurs when **k** = 0. For simplicity, we define: *β*^2^ = 2*m*_qw,e(h)_*E*_*e*(h)_ℏ^−2^ and *α*^2^ = 2*m*_b,e(h)_(*V*_e(h*)*_ – *E*_e(h)_)ℏ^−2^. The effective masses of electrons and holes are assumed to be the same for the quantum well ($m_{{\mathrm{qw}}} = m_{{\mathrm{qw}},{\mathrm{e}}} = m_{{\mathrm{qw}},{\mathrm{h}}}$) and barrier ($m_{\mathrm{b}} = m_{b,{\mathrm{e}}} = m_{{\mathrm{b}},{\mathrm{h}}}$). We apply literature values for the effective mass in the metal halide layer^57^ (*m*_qw_ = 0.1*m*_0_, where *m*_0_ is the rest mass of an electron) and the barrier layer^64^ ($m_{\mathrm{b}} = m_{{\mathrm{qw}}}/0.4$). The barrier height (*V*_e(h)_) for the electrons (holes) is an expression of the bandgap of the formamidinium layers that separate metal halide layers. For simplicity, we assume $V_{\mathrm{e}} = V_{\mathrm{h}}$. The expression for *γ* is modified from the classic KP model ($\gamma = \alpha /\beta$) to take into account the difference in effective mass of the electrons (holes) in the quantum well and barrier layers: $\gamma = {\upalpha}m_{{\mathrm{qw}},{\mathrm{e}}({\mathrm{h}})}/\beta m_{{\mathrm{b}},{\mathrm{e}}({\mathrm{h}})}$.

PL shows discrete miniband transitions from the optical bandgaps of a mixture of FA_n+1_Pb_n_I_3n+1_ thicknesses (*n*) that increase as the RH increases (Fig. 6b). As the RH increases to 80%, the PL peak shifts from 2.28 eV to 1.71 eV. It is notable that the PL is tuned in the visible region over a 0.56 eV window by simply varying the RH. The PL is quenched upon reaching 82% RH, which is consistent with the transition to δ-FAPbI_3_^28^. We successfully reproduce our experimental PL by numerically solving Eq. 2 for $E_{{\mathrm{e}}({\mathrm{h}})}$ to produce $E_{{\mathrm{g}},2{\mathrm{D}}}$ as a function of quantum well width ($L_{{\mathrm{qw}}}$) (Fig. 6c). The thickness of a monolayer in FA_n+1_Pb_n_I_3n+1_ is 0.624 nm. The KP-like model nicely reproduces our optical bandgap data determined from PL measurements for *n* > 2. A potential barrier height of $V_{{\mathrm{e}}({\mathrm{h}})}$ = 1.2 eV best fits the data, which is a reasonable bandgap for a FAI salt layer ($E_{{\mathrm{g}},{\mathrm{FAX}}} = E_{{\mathrm{g}},3{\mathrm{D}}} + e\left( {V_{\mathrm{e}} + V_{\mathrm{h}}} \right)$ = 3.92 eV) between the lead halide sheets. The data is captured by varying the barrier height between 0.8 and 1.6 eV. We posit the barrier height will be affected by the presence of water vapor interacting with the system. The model is also in good agreement with previous work on layered MHP materials, where butyl groups separate methylammonium lead halide layers^43^. The monolayer (*n* = 1) case is not described well by our model, in addition to data from Kanatzitis^43,65^ and others^66^. The deviation is known to occur due to the increasing dielectric confinement, which increases the exciton binding energy in *n* = 1 materials^67–69^. Intuitively, the bandgap of a lead iodide monolayer is no longer represented well by the bulk properties of α-FAPbI_3_.

We expect sizable differences in the photoconductivity of each phase as the FA_n+1_Pb_n_I_3n+1_ grains transition between 2D FA_n+1_Pb_n_I_3n+1_, 3D α-FAPbI_3_, and 1D δ-FAPbI_3_ based on differences in excitonic character and binding energy. We measure these differences using contactless flash-photolysis time-resolved microwave conductivity (fp-TRMC)^70,71^ by photoexciting composite FA_n+1_Pb_n_I_3n+1_ films at 450 nm and at the lowest possible laser fluences (*ca*. 10^11^–10^12^ photons cm^−2^) to obtain satisfactory signal-to-noise while avoiding higher-order recombination effects associated with high charge densities (Supplementary Fig. 16). Indeed, we observe a significant change in the yield-mobility product (*ϕΣμ*) when comparing films exposed to 20% RH (yellow) and those to 70–78% RH (orange and brown). The orange and brown phases demonstrate *ca*. 5–6 times higher *ϕΣμ* values compared to the yellow phase (Table 1, Supplementary Table 1), which is likely due in part to increases in charge yield (*ϕ*) as the exciton binding energy decreases for more 3D connected (higher *n*) domains. We believe electron and hole mobilities (represented by *Σμ*) increase concomitantly with charge yield^72^ and argue the *ϕΣμ* values of the orange phase are closer to the brown phase likely due to an appreciable density of *n* > 4 domains that dominate the fp-TRMC signal over smaller (*n* = 1–3) layers. Putting limits on the sum of the hole and electron mobilities for these compounds, we estimate that values of 0.003–0.3 and 0.019–1.9 cm^2^ V^−1^ s^−1^ are possible for yellow and brown phases, respectively, assuming charge yields between 100 and 1%.

**Table 1:** Average *ϕΣμ* values measured by fp-TRMC at 450 nm.

RH (%)	Color	*n*	*ϕΣμ* (cm^2^ V^−1^ s^−1^)
20 ± 3	Yellow	1–2	0.003 ± 0.002
70 ± 3	Orange	2–4	0.014 ± 0.002
78 ± 3	Brown	>4	0.019 ± 0.008

*ϕΣμ* values obtained for the brown (most photoconductive) phase were comparable to those obtained in other works. (BA)_2_(MA)_n-1_Pb_n_I_3n+1_ (*n* = 3–4) was measured to have *ϕΣμ* = 0.03–0.04 cm^2^ V^−1^ s^−1^ ^73^. However, these films were measured at up to 2 orders of magnitude higher fluence (*ca*. 10^13^ photons cm^−2^) than the FA_n+1_Pb_n_I_3n+1_ composite films measured here (*ca*. 10^11^ photons cm^−2^). As such, the values for BA-based 2D Ruddlesden-Popper MHPs obtained from that work are likely higher than reported since the low fluence regimes, where *ϕΣμ* values exhibit little-to-no fluence-dependence, were not measured. Indeed fp-TRMC measurements on (BA)_2_(MA)_n−1_Pb_n_I_3n+1_ (n = 4–5) by Gélvez-Rueda et al.^72^ at fluences similar to this work demonstrated *ϕΣμ* values *ca*. 10–30 cm^2^ V^−1^ s^−1^, over three orders of magnitude larger than the FA_n+1_Pb_n_I_3n+1_ MHPs studied here. Measurements on (MA)_2_Pb(SCN)_2_I_2_ (*n* = 1)^74^ and (BPEA)_2_(MA)Pb_2_I_7_ (*n* = 2)^75^ yield *ϕΣμ* values *ca*. 1 cm^2^  V^−1^ s^−1^ which is ca. 50 times greater than FA_n+1_Pb_n_I_3n+1_. A less dramatic difference was found when comparing the range of amplitude-weighted average free charge lifetimes measured for the brown phase (*ca*. 20–90 ns) to (MA)_2_Pb(SCN)_2_I_2_ (*n* = 1, *ca*. 100 ns)^74^ and (BA)_2_(MA)_n-1_Pb_n_I_3n+1_ (*n* = 4–5, *ca*. 100–200 ns)^72^. Our scaffold-impregnated films likely have random crystallite orientation, which will lower our *ϕΣμ* values relative to some of the literature discussed here^73^. Only free charges within crystallites oriented parallel to our linearly polarized microwave probe will absorb appreciably. The variation of free charge lifetimes (and to some degree *ϕΣμ*) for the brown phase on a film-to-film basis could be due to differences in the density of crystallites and crystallite size distribution based on modest differences in film processing conditions, such as scaffold thickness and Al_2_O_3_ nanoparticle size dispersity used in the scaffold. We were not able to measure *ϕΣμ* values of the white/colorless phase, as the lowest fluences were sufficient to convert this phase into the brown phase via heat-induced dehydration mechanism (Supplementary Fig. 17) that we have observed at temperatures as low as 35 °C (Supplementary Fig. 12).

## Discussion

We synthesized layered FA-based MHPs of the general formula FA_n+1_Pb_n_X_3n+1_ (X = I, Br) and their mixed-halide compositions and show reversible multicolor chromism based on two different stimuli: solvent vapor and temperature. We design composite FA_n+1_Pb_n_X_3n+1_ films by synthesizing FA_n+1_Pb_n_X_3n+1_ domains adjacent to a FAX “reservoir” composed of excess FAX salt that allows FAX salt pairs to shuttle between FA_n+1_Pb_n_X_3n+1_ domains and the FAX reservoir. The interactions between each species results in a structural equilibrium between 2D FA_n+1_Pb_n_X_3n+1_ layers, 3D α-FAPbX_3_ domains, and 1D δ-FAPbI_3_ domains that can be controlled through modulating the strength and number of H-bonds between the reservoir, MHP, and solvent vapor. Unlike previously reported chromic MHPs that only switch between a single dark and a single transparent phase, these films reversibly switch between multiple colors including yellow, orange, red, brown, and white/colorless. The optical transitions are captured by a Kronig–Penney-like model that describes photoluminescence of the material as metal halide octahedra layers separate and coalesce into superlattices of varied thickness. Our work will enable a new generation of functional materials that couple tunable and reversible chromism with the extraordinary optoelectronic properties of MHP materials.

## Methods

### Materials

Aluminum oxide nanoparticles (Al_2_O_3_, <50 nm particle size, 20 wt% in isopropanol) and dimethyl sulfoxide (DMSO, anhydrous, ≥99.9%) were purchased from Sigma-Aldrich; lead(II) iodide (PbI_2_, 99.99%) from TCI; formamidinium iodide (FAI) from GreatCell Solar Materials.

### FA_2_PbI_4_ powder preparation

Powders were prepared by weighing a 4:1 molar ratio of FAI:PbI_2_ in a nitrogen glovebox, sealing in a hardened stainless-steel vial, then ball-milling in a SPEX high energy ball mill for 60 minutes. The vial was then transferred back to the glovebox for recovery of the milled powders. Powders were not exposed to air/humidity until hygrochromic tests were conducted.

### FA_n+1_Pb_n_X_3n+1_ composite film preparation

Glass substrates (25 mm × 25 mm × 1 mm) were sonicated in isopropanol (IPA) for 10 min and blown dry with N_2_. The substrates were then treated in a UV-ozone cleaner for 10 min before spin-coating 200 µL of 16 wt% Al_2_O_3_ nanoparticles (20–40 nm) in IPA at 3000 rpm for 30 s. The resulting film was annealed at 150 °C for 5 min followed by 500 °C for 25 min. This process yielded a 1.58 ± 0.02 μm-thick Al_2_O_3_ scaffold. The Al_2_O_3_ NP precursor can be diluted with IPA to yield thinner films as shown in Supplementary Fig. 18. Next, 75 µL of a precursor solution containing 3 M FAX and 0.75 M PbX_2_ (4:1 FAX:PbX_2_) in DMSO was spin-coated at 4000 rpm for 30 s followed by annealing at 60 °C for 10 min. We note here that precursor concentration must be optimized for a given scaffold thickness. Optimal precursor concentrations based on PbX_2_ ([FAX] = 4[PbX_2_]) for a given scaffold thickness are as follows: 0.7–1 M PbX_2_ for 1.0–1.6 µm thick, 0.4–0.6 M PbX_2_ for 600–1000 nm thick, and 0.3–0.4 M PbX_2_ for 300–600 nm thick. Both spin-coating and annealing were performed in air while maintaining the relative humidity (RH) between 40 and 45%. The substrates were stored in a drawer (<20% RH) or desiccator in air until needed. See Supplementary Note 2 for a discussion on the impact of processing conditions on composite FA_n+1_Pb_n_X_3n+1_ film formation.

### Humidity control

Humidy-controlled experiments were performed in a glovebox equipped with a humidity controller connected to a humidity sensor (error of ± 3%), humidifier, and dehumidifier unless noted elsewhere. The controller maintains the humidity with an accuracy of ± 0.1–0.3% The humidity controller was calibrated to saturated KBr in water, which exhibits a relative humidity (RH) of 81.67 ± 0.21% at 20 °C^76^. We reported RH throughout this study; however, RH is different at sea level compared to high altitudes (NREL, Golden, CO, this study). We converted the RH’s reported in this study to absolute humidities (AH’s) using 25 °C and 630 mmHg as a typical barometric pressure of NREL in Golden, CO (Supplementary Table 2).

### Optical characterization

Absorbance was collected with a photodiode array Hewlett-Packard 8453 UV-vis spectrometer with Al_2_O_3_ scaffold and glass absorbance subtracted from all spectra. Steady-state PL measurements were taken using a home-built system. Samples were excited using a Thorlabs fiber-coupled 405 nm light-emitting diode (LED) pulsed at 10 Hz using a Thorlabs DC2200 LED driver. Visible detection was made using an Ocean Optics OceanFX spectrometer. Spectra were stitched using a LabVIEW program developed in-house. Detector calibration was done using an Ocean Optics HL-2000-HP blackbody lamp. Prior to data acquisition, the substrate was sealed into an air-tight optical holder filled with the desired RH. Optical measurements were collected on films prepared with a 326 ± 22 nm thick Al_2_O_3_ NP scaffold.

### Structural characterization

In situ X-ray diffraction data was collected at the Stanford Synchrotron Radiation Light Source (SSRL) at beamline 11-3. The samples were measured at an incident angle of 3 degrees and an incident X-ray wavelength of 0.9744 Å. A Rayonix MX225 2D detector was used to collect data, and a LaB_6_ standard used to calibrate the data. The RH in the sample chamber was alternated between 35% and 82% RH using a commercial room humidifier powered by a humidity controller, which was connected to a humidity sensor within the sample chamber. The humidity in the sample chamber was reduced by flowing helium gas through the chamber. Once 35% RH was reached, the humidity was increased by manually reducing the He flow until the humidity stabilized at 82% RH. The data collection were continuous except for brief interruptions for changing the sample chamber connection between the humidifier and the He. The integration time per measurement was 30 s. The data was integrated using GSAS-II^77^. 2*θ* values were calculated by converting *Q* data relative to Cu Kα (1.5406 Å, 8.04 eV). Scherrer analysis was performed using a κ value of 0.9 and FWHM values with error bars obtained by fitting peaks to a Voigt function. Non-synchrotron WAXS of composite films was collected in air using a Bruker D8 Discover diffractometer with GADDS 4-circle detector (General Area Detector Diffraction System) and Cu Kα (1.5406 Å, 8.04 eV) radiation. WAXS of powder was collected in air using a Rigaku Smartlab diffractometer using Cu Kα (1.5406 Å, 8.04 eV) radiation. Fast scans (ca. 2 min) were done before and after a long scan (ca. 30 min) to verify no change in the material due to beam exposure. Interlayer spacing of butylammonium, phenylethylammonium, and hexylammonium was calculated using Jmol by performing an energy minimization of the molecule, measuring the longest hydrogen–hydrogen distance, and then multiplying this distance by 2 based on the assumption that the interlayer spacing is composed of two end-to-end molecules.

### DRIFTS measurements

A Bruker Alpha FTIR spectrometer outfitted with a diffuse-reflectance infrared Fourier transform spectroscopy (DRIFTS) attachment was used in the study, and all measurements were performed in a humidity glovebox as described above. Samples were prepared as described above except Au-coated Si was used instead of glass as the substrate. DRIFTS data were collected between 350 and 4000 cm^−1^ with a resolution of 2 cm^−1^ and was atmosphere corrected. RH and temperature data were collected using a data logger with points collected every second. Each DRIFTS spectrum was collected over 45 s, so 45 individual RH and temperature points were averaged to determine the average RH and temperature of each spectrum. The RH was increased at a rate of 3.2 ± 0.2% min^−1^ and decreased at a rate of 1.9 ± 0.3% min^−1^ during data acquisition.

### fp-TRMC measurements

Our flash-photolysis time-resolved microwave conductivity (fp-TRMC) measurement system and methods has been described in detail elsewhere^70,71^. Photoexcitation was accomplished using a Nd:YAG (Spectraphysics Quanta Ray SP Pro 230-30H) laser with 9 W of 355 nm at 30 Hz to pump an OPO (Spectraphysics GWU PremiScan ULD/500) with output over the range of 410–2500 nm with 7 ns pulses (ca. 3 W output, varies by wavelength). Blank quartz substrates and quartz substrates with only the alumina scaffold were used to gauge any background contributions to the fp-TRMC transients and showed no appreciable response. All samples were excited at 450 nm with fluences in the range of *ca*. 1 × 10^11^ to 2 × 10^15^ photons cm^−2^ depending on the strength of the sample response and phase stability during illumination. A 399 nm long pass filter and 700 nm short pass filter were used to cut out residual 355 and 1064 nm light from the optical parametric oscillator, respectively. Maintaining the RH levels necessary to stabilize the brown phase during measurements was achieved by sealing samples in a leak-resistant microwave cavity within the humidified glovebox described previously. At higher fluences, phases held at higher RH tended to revert back to phases at lower ones, possibly due to heat-driven dehydration of the film (Supplementary Fig. 17). For this reason, the white/colorless phase could not be measured at even the lowest fluences. Phase purity was assessed by monitoring the steady-state photoluminescence for films in-situ during fp-TRMC measurements (e.g., see PL data in Supplementary Fig. 19) using a camera lens to focus emitted photons from the microwave cavity into an optical fiber that was coupled to a Princeton Instruments SpectraPro 2500i spectrometer with a liquid-nitrogen cooled CCD detector. A 500 nm long pass filter was used to filter out excitation light (450 nm) and photoluminescence spectra were typically acquired by averaging 60 seconds to monitor phase stability on a minute-to-minute basis during measurements. PL spectra were not corrected by a calibration lamp. The yellow phase was the only sample for which the entire fluence range was measured. Typical fluences for orange and brown samples were ca. 1–20 × 10^11^ photons/cm^2^. Most transients were acquired for 30,000 shots or until S/N = 10. For some yellow and orange samples with lower S/N at low fluences, up to 60,000 shots were acquired. The typical fraction of absorbed photons at 450 nm for yellow, orange, and brown phases were 99%, 99%, and 96%, respectively. Transient fitting was done using a custom global fitting routine in Igor Pro 8 using a biexponential fit. *ϕΣμ* values at each fluence were extracted by summing the amplitudes of the optimized fit coefficients. For each sample, an average of *ϕΣμ* over the lowest four-to-five fluences (i.e., regime where *ϕΣμ* shows little-to-no fluence dependence) were taken to represent the average yield-mobility product for that sample. These values were then further averaged over three samples to obtain the values and uncertainties reported here.

## Supplementary information

Supplementary Information

Peer Review File

Supplementary Movie 1

## Data Availability

The data that support the findings of this study are available from the corresponding author upon reasonable request.

## Code availability

Mathematica code used to model FA_n+1_Pb_n_I_3n+1_ optical properties is available from the corresponding author upon reasonable request.
