# Supplementary material for: Reversible multicolor chromism in layered formamidinium metal halide perovskites
Source: Nat Commun. 2020 Oct 16;11:5234. doi: 10.1038/s41467-020-19009-z (PMC7568568; doi:10.1038/s41467-020-19009-z)
Supplement: Supplementary file 1 — Supplementary Information [file 41467_2020_19009_MOESM1_ESM.pdf]

Supplementary Information

Reversible multicolor chromism in layered formamidinium metal halide perovskites

Bryan A. Rosales,[1] Laura E. Mundt,[2] Taylor G. Allen,[1] David T. Moore,[1] Kevin J. Prince,[1,3] Colin A. Wolden,[3,4] Garry Rumbles,[1,5] Laura T. Schelhas,[2] and Lance M. Wheeler[*,1]

[1]Center for Chemistry and Nanoscience, National Renewable Energy Laboratory, 15013 Denver West Parkway, Golden, Colorado 80401, USA.

[2]SLAC National Accelerator Laboratory, 2575 Sand Hill Road, Menlo Park, California 94025, USA.

[3]Department of Chemical and Biological Engineering, Colorado School of Mines, Golden, Colorado 80401, USA.

[4]Material Science Program, Colorado School of Mines, Golden, Colorado 80401, USA.

[5]Renewable and Sustainable Energy Institute, Department of Chemistry, University of Colorado, Boulder, Colorado 80309, USA

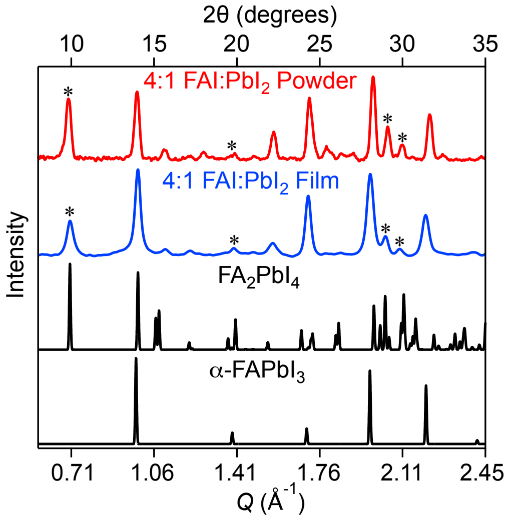

Supplementary Figure 1 | $FA_{n+1}Pb_nX_{3n+1}$ powder prepared by ball-milling 4:1 FAX:PbX_2. Wide angle X-ray scattering (WAXS) comparison of $FA_{n+1}Pb_nI_{3n+1}$ powder and films.

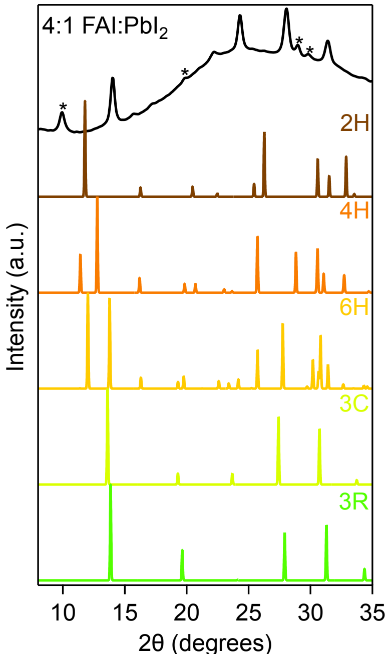

Supplementary Figure 2 | Comparison of $FA_{n+1}Pb_nI_{3n+1}$ film wide angle X-ray scattering (WAXS) data to structures commonly observed during formation of FA-based halide perovskites. Standard patterns were obtained from ref. [1] and modified to include only FA^+, Pb^{2+}, and I^- ions by multiplying the unit cell volume by the appropriate ionic radius ratio. We note that 2H corresponds to δ-$FAPbI_3$, 3C corresponds to single-crystalline α-$FAPbI_3$, and 3R corresponds to thin-film α-$FAPbI_3$.

Supplementary Note 1. Derivation of '*q*' index.

$$FA_{n+1}Pb_nX_{3n+1} \leftrightarrow FA_{n+1-q}Pb_nX_{3n+1-q} + qFAX \quad (1)$$

$$n = 1, 2, 3, \dots \infty; \; q = \frac{1}{n(n+1)}$$

At a given value of 'n', the amount of FAX relative to Pb can be represented as:

$$\frac{n+1}{n}$$

When we transition to a larger 'n' value, the amount of FAX relative to Pb can be modified to:

$$\frac{(n+1)+1}{n+1}$$

'*q*' is the amount of FAX gained or lost by transitioning between 'n' values. Therefore,

$$q = \frac{(n+1)+1}{n+1} - \frac{n+1}{n}$$

$$= \left(\frac{n}{n}\right)\frac{(n+1)+1}{n+1} - \left(\frac{n+1}{n+1}\right)\frac{n+1}{n} = \frac{n(n+2)}{n(n+1)} - \frac{(n+1)^2}{n(n+1)} = \frac{n(n+2)-(n+1)^2}{n(n+1)}$$

$$= \frac{n^2+2n-n^2-2n-1}{n(n+1)} = -\frac{1}{n(n+1)}$$

Supplementary Note 2. Impact of processing conditions on composite $FA_{n+1}Pb_nX_{3n+1}$ film formation.

We found the degree of hygrochromism depends significantly on the processing conditions including annealing temperature, required use of a scaffold, precursor concentration, use of a large FAX excess relative to PbX_2 (> 3 : 1 FAX : PbX_2), the ambient humidity when spun or annealed, and storage conditions. The following discusses the effects of each processing condition using $FA_{n+1}Pb_nI_{3n+1}$/water vapor as a model system.

Annealing these films at temperatures > 60 °C irreversibly darkens the yellow film to orange (100-160 °C) and brown (160-180 °C), with the brown film exhibiting absorbance characteristic of bulk α-$FAPbI_3$ (Supplementary Figure 3). High temperatures likely drive off DMSO and H_2O, which play a vital role in the switching mechanism. However, the films are still hygrochromic until they turn brown, but they will have reduced color range when annealed at higher temperatures.

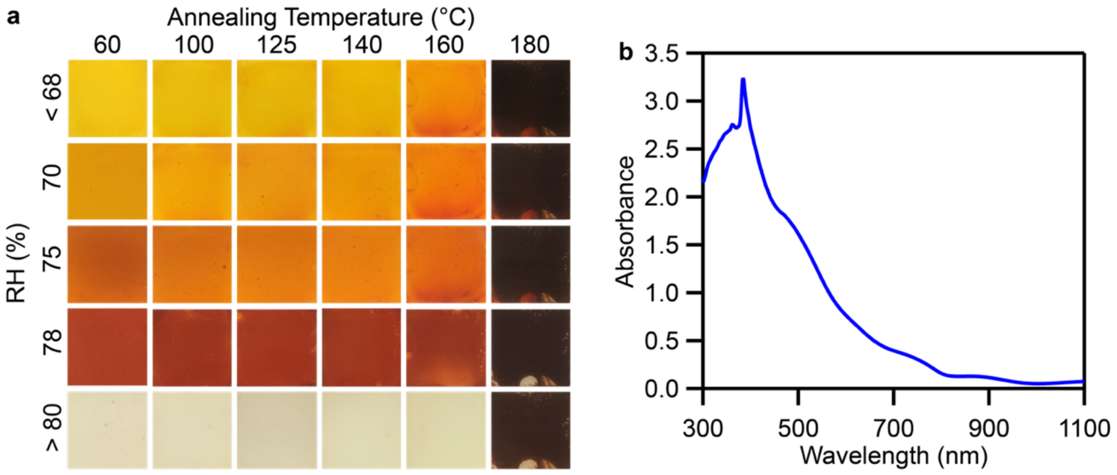

Supplementary Figure 3 | Effect of higher annealing temperatures on chromic properties. a Representative visual images of composite $FA_{n+1}Pb_nI_{3n+1}$ films annealed at higher temperatures and exposed to increasing relative humidity (RH). **b** Absorbance of film after heating at 180 °C followed by cooling to 20 °C.

A scaffold is necessary for the observed hygrochromic properties to occur, as no chromism is observed without a scaffold even though the phases determined by WAXS are identical (Supplementary Figure 4). However, WAXS peaks of films grown on ITO are much sharper, which suggests the film exhibits larger domains.

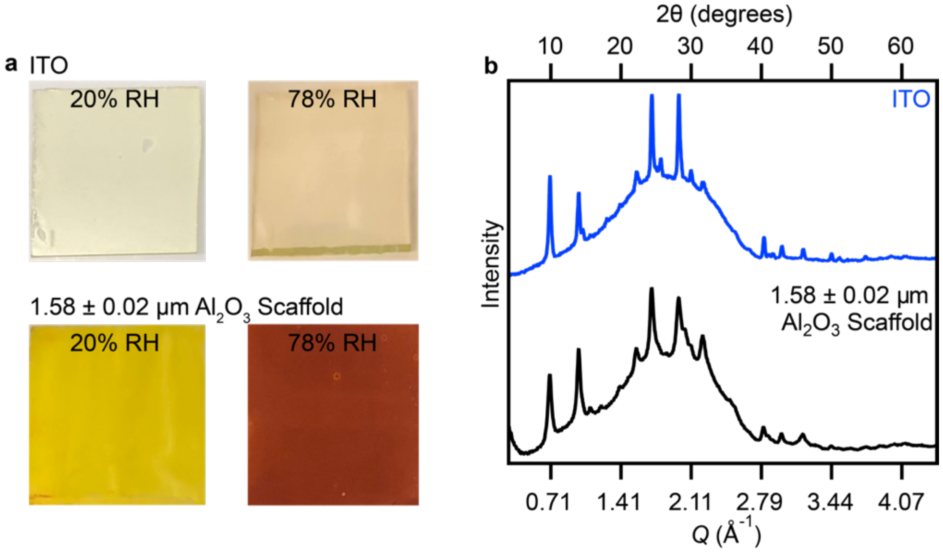

Supplementary Figure 4 | Effect of scaffold on chromic properties. a Visual images showing no hygrochromic behavior when $FA_{n+1}Pb_nI_{3n+1}$ films are prepared on ITO without a scaffold (top images) and hygrochromic behavior when prepared on a 1.58 ± 0.02 μm Al_2O_3 nanoparticle scaffold (bottom images). Films were exposed to varying relative humidity (RH) under static conditions (no flow, jar of saturated salt solution). **b** Wide angle X-ray scattering (WAXS) comparison of $FA_{n+1}Pb_nI_{3n+1}$ films prepared on both substrates. The 2θ axis in **b** is relative to Cu Kα (1.5406 Å, 8.04 eV) radiation and was calculated from $Q = 4\pi\sin(\theta)/\lambda$ where λ is the excitation wavelength.

The concentration of the precursor solution must be optimized to a given scaffold thickness for complete hygrochromic properties to occur. Optimal precursor concentrations based on [PbI_2] ([FAI] = 4[PbI_2]) for a given Al_2O_3 NP scaffold thickness are as follows: 0.7-1 M PbI_2 for 1.0-1.6 µm thick, 0.4-0.6 M PbI_2 for 600-1000 nm thick, and 0.3-0.4 M PbI_2 for 300-600 nm thick. If the precursor concentration is too high, a thick perovskite film is formed on top of the scaffold and the film does not turn brown (Supplementary Figure 5a) similar to when no scaffold is used (Supplementary Figure 4a). If the precursor concentration is too low, the resulting film is much darker with optical properties characteristic of bulk α-$FAPbI_3$ (Supplementary Figure 5) and with suppressed reversible hygrochromic properties. When exposed to high RH, the darker films irreversibly turn into white/colorless δ-$FAPbI_3$ except upon further heating to > 175 °C. We also note that DRIFTS shows our scaffold is hydroxyl-terminated (Supplementary Figure 6). All these results suggest that the scaffold assists in the switching mechanism likely by acting as a "moisture highway" that transports moisture along its hydrophilic surface (Supplementary Figure 7).

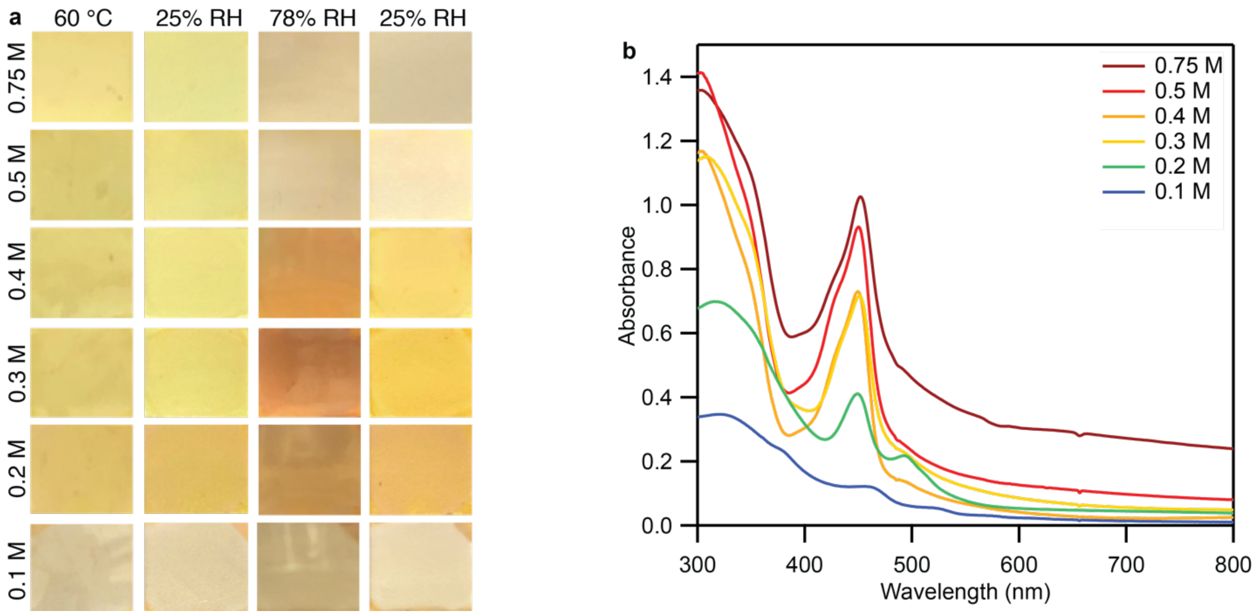

Supplementary Figure 5 | Effect of precursor concentration on chromic properties. a Representative images showing the influence of [PbI_2] ([FAI] = 4[PbI_2]) precursor concentration on the hygrochromic switching behavior when a 326 ± 22 nm thick Al_2O_3 nanoparticle scaffold is used. Hygrochromic properties are only observed at precursor concentrations between 0.3-0.4 M. The precursor concentration must be matched with a specific scaffold thickness for hygrochromic properties to occur. **b** Absorbance collected of as-prepared films exposed to 25% RH.

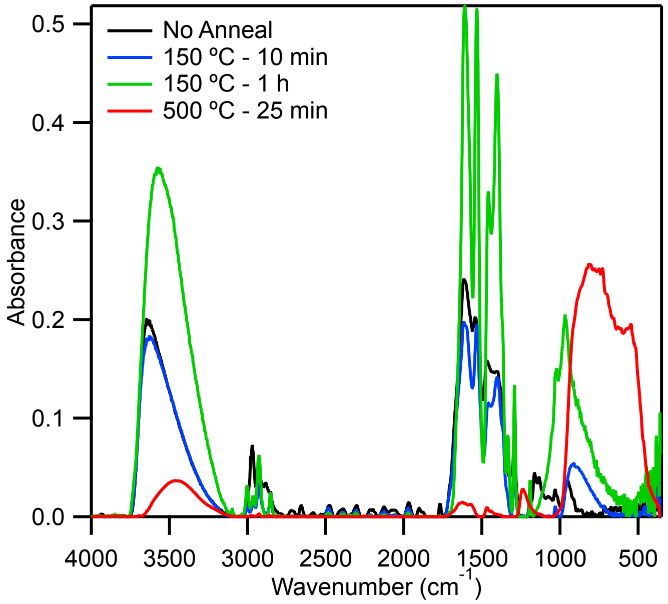

Supplementary Figure 6 | Surface termination of Al_2O_3 nanoparticle scaffold at different annealing temperatures. Diffuse-reflectance infrared Fourier transform spectroscopy (DRIFTS) collected of Al_2O_3 nanoparticle scaffolds annealed at various temperatures. Upon annealing to 500 °C, all hydrophobic ligands are burned off and the surface is hydroxyl-terminated.

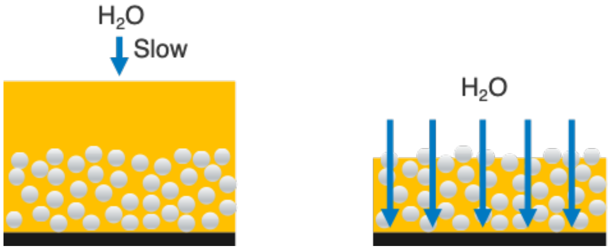

Supplementary Figure 7 | Dependence of moisture transport on scaffold and metal halide perovskite thickness. Illustration of an overfilled and perfectly filled Al_2O_3 nanoparticle scaffold.

The color of the as-prepared film and the degree of hygrochromic properties can be manipulated by varying the FAX concentration (Supplementary Figure 8). No hygrochromic properties were observed if the FAI:PbI_2 ratio is less than 3.0, which shows that enough FAI must be introduced to form both the "FAI reservoir" and $FA_{n+1}Pb_nI_{3n+1}$. Interestingly, the color of the as-prepared film lightens and the range of achievable colors increases as the FAI:PbI_2 ratio increases. Larger amounts of FAI push the equilibrium to thinner layers (smaller n) because more FAI can be incorporated between the layers. This is explicitly shown by WAXS (Supplementary Figure 8b) in which only the film fabricated with a FAI:PbI_2 ratio of 4.0 observes the peak corresponding to FA_2PbI_4 (n = 1) after annealing at 60 °C for 10 min.

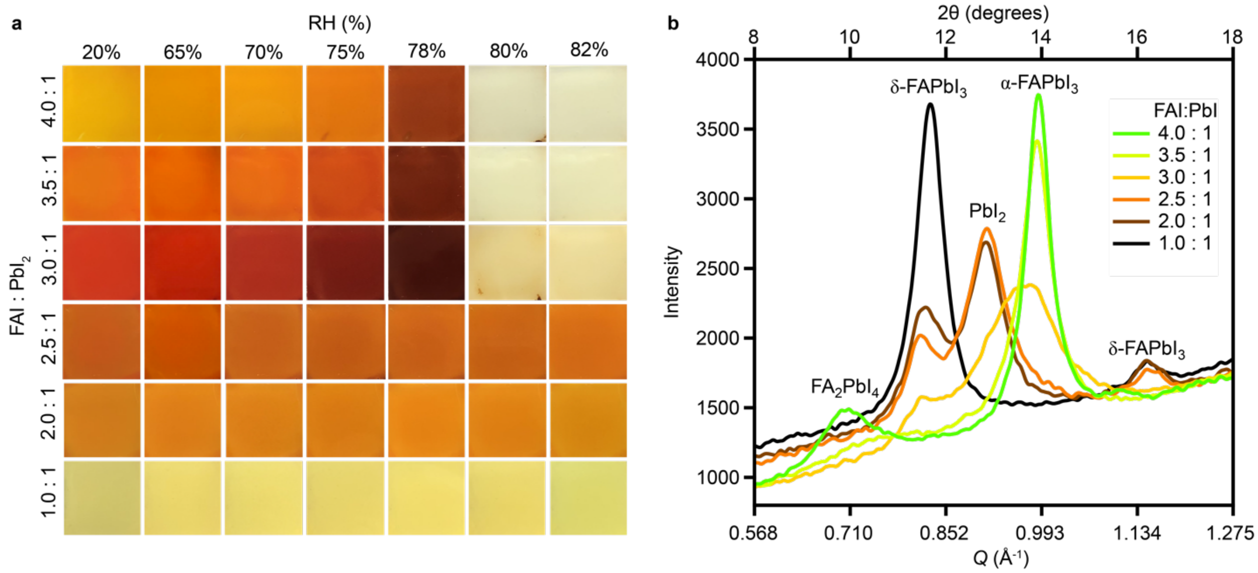

Supplementary Figure 8 | Effect of FAI:PbI_2 on chromic properties. a Representative images and **b** wide angle X-ray scattering (WAXS) data that show the dependence of hygrochromic switching behavior on the FAI:PbI_2 ratio used during film fabrication. The FAI:PbI_2 ratio was varied while [PbI_2] = 0.75 M was held constant in dimethyl sulfoxide (DMSO). Films were spun onto a 1.58 ± 0.02 µm Al_2O_3 nanoparticle scaffold and were annealed at 60 °C for 10 min.

Hygrochromic $FA_{n+1}Pb_nX_{3n+1}$ films must be spun and annealed in the presence of ~40% RH to yield optimal hygrochromic properties (Supplementary Figure 9). If the films are both spun and annealed in a N_2 glovebox (0% RH) or at 15% RH in air, only incomplete yellow to orange hygrochromism is observed. Yellow to orange hygrochromism is also observed if the film is spun in a N_2 glovebox (0% RH) but annealed at 40% RH in air. Yellow to orange to brown hygrochromism with significantly slowed transition to white/colorless is observed when the film is spun at 40% RH in air but annealed in a N_2 glovebox (0% RH). Complete hygrochromism (Supplementary Figure 9a) is only observed if the film is both spun and annealed at 40% RH in air. Absorbance collected on films prepared by all of the above methods are similar with no systematic change. However, WAXS shows significant texturing in films exhibiting only yellow to orange hygrochromism. Thus, some moisture is necessary during film preparation for complete hygrochromic properties.

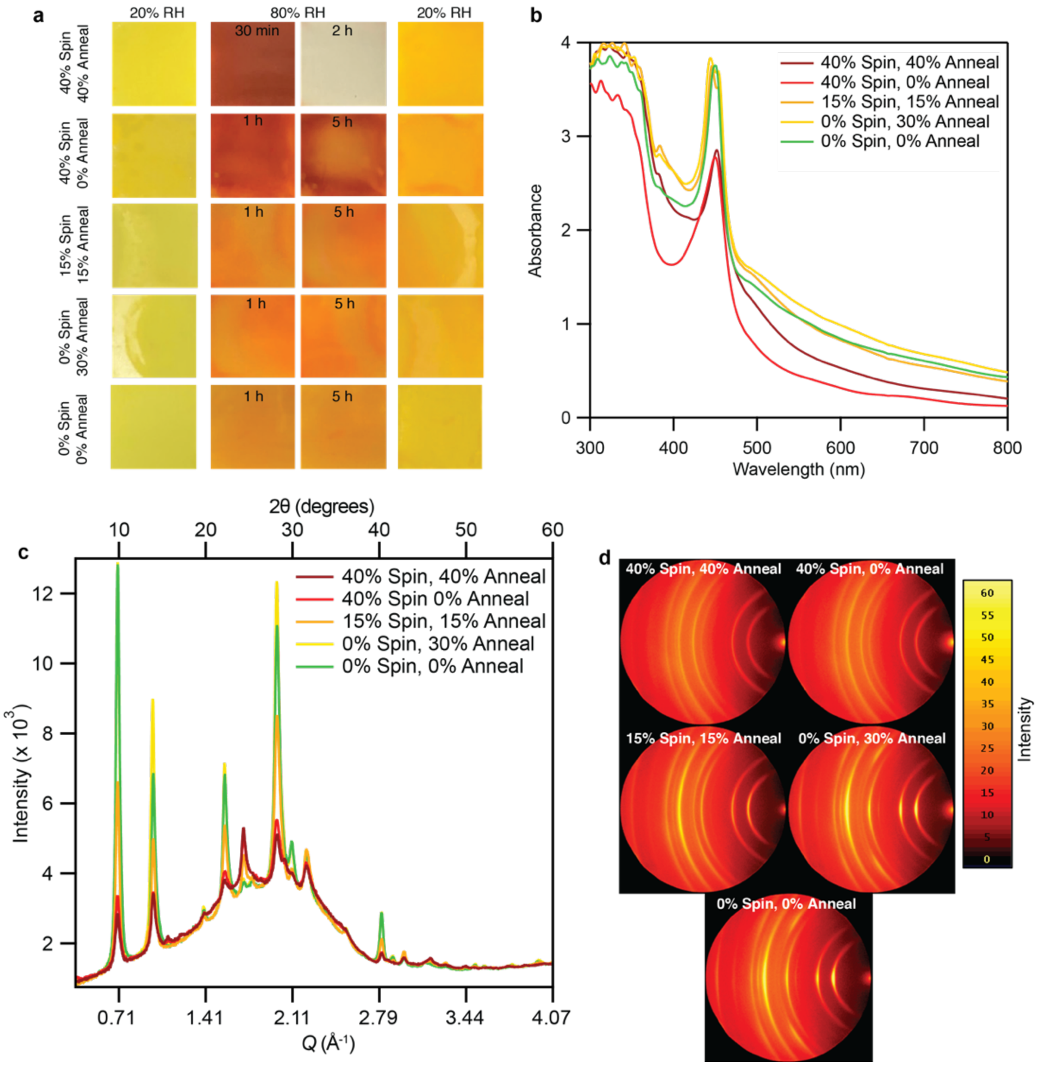

Supplementary Figure 9 | Effect of ambient humidity during film fabrication on chromic properties. a Representative visual images, **b** absorbance, **c** wide angle X-ray scattering (WAXS), and **d** 2D WAXS images of composite $FA_{n+1}Pb_nI_{3n+1}$ films prepared by various methods and then exposed to 80% static relative humidity (RH) (no flow, jar of saturated salt solution) for various times.

Lastly, complete hygrochromic properties are susceptible to prolonged dry-air flow. If the films are stored in a flowing N_2 box, in a fume hood, or on the counter of a lab with high airflow, the films irreversibly darken to brown over several hours to days depending on the flow rate and RH. These conditions remove volatile DMSO and H_2O in the film, which are necessary for switching to occur. Therefore, we recommend storage of the films in a closed container (lab drawer, jar, etc…). These hygrochromic films are stable under these storage conditions for several months.

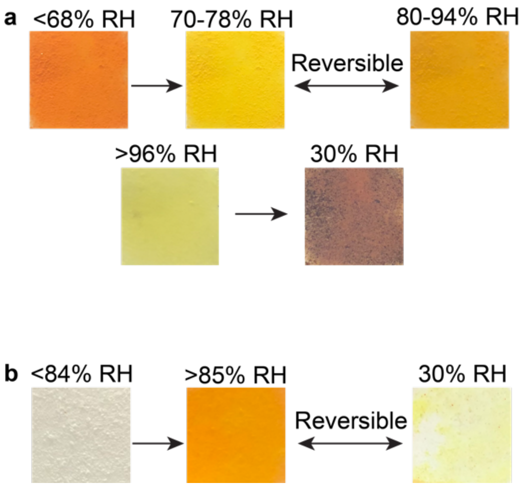

Supplementary Figure 10 | Hygrochromism in $FA_{n+1}Pb_nX_{3n+1}$ powder prepared by ball-milling 4:1 FAX:PbX_2. Representative visual images demonstrating hygrochromism in **a** $FA_{n+1}Pb_nI_{3n+1}$ and **b** $FA_{n+1}Pb_nBr_{3n+1}$ powder.

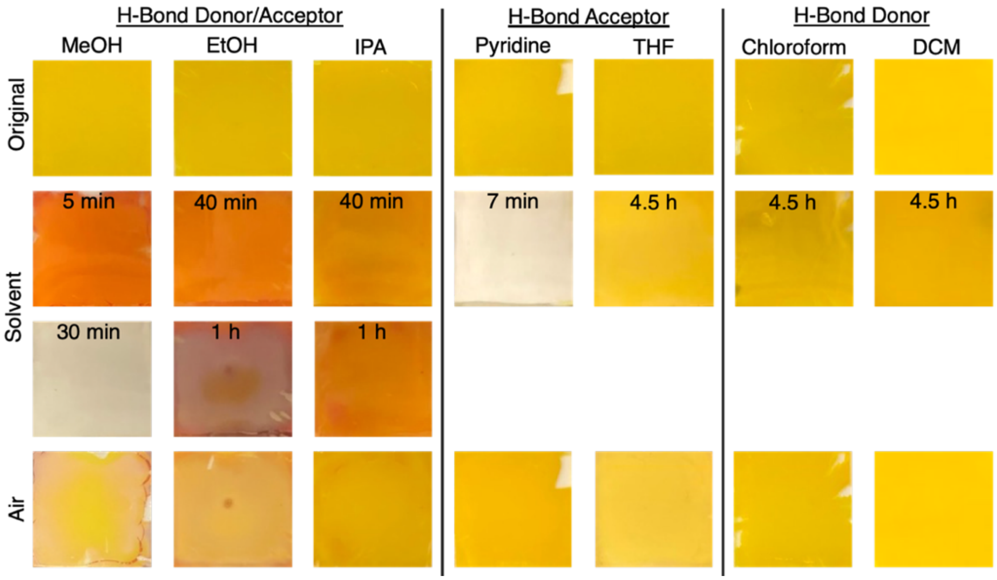

Supplementary Figure 11 | Dependence of chromic properties on the solvent vapor identity. Composite $FA_{n+1}Pb_nI_{3n+1}$ films exposed to various solvent vapors under static conditions (no flow, jar with each solvent). Multiple colors are obtained if the solvent vapor is an H-bond donor.

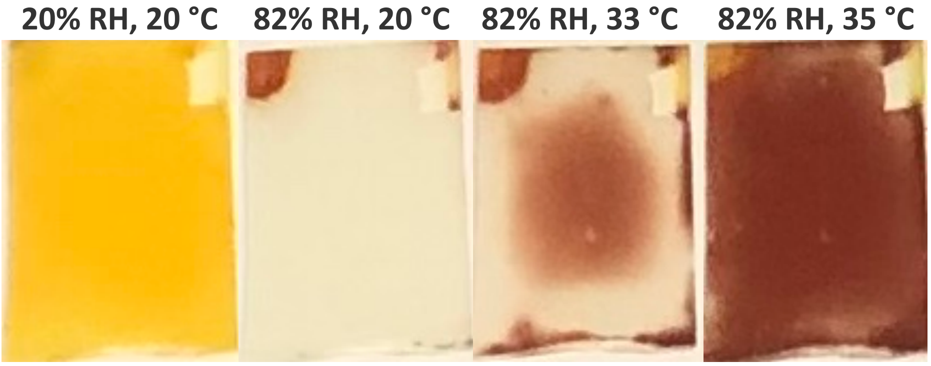

Supplementary Figure 12 | Thermochromic behavior. A $FA_{n+1}Pb_nI_{3n+1}$ film bleached upon exposure to 82% relative humidity (RH) can be converted to the brown color by heating to temperatures as low as 35 °C.

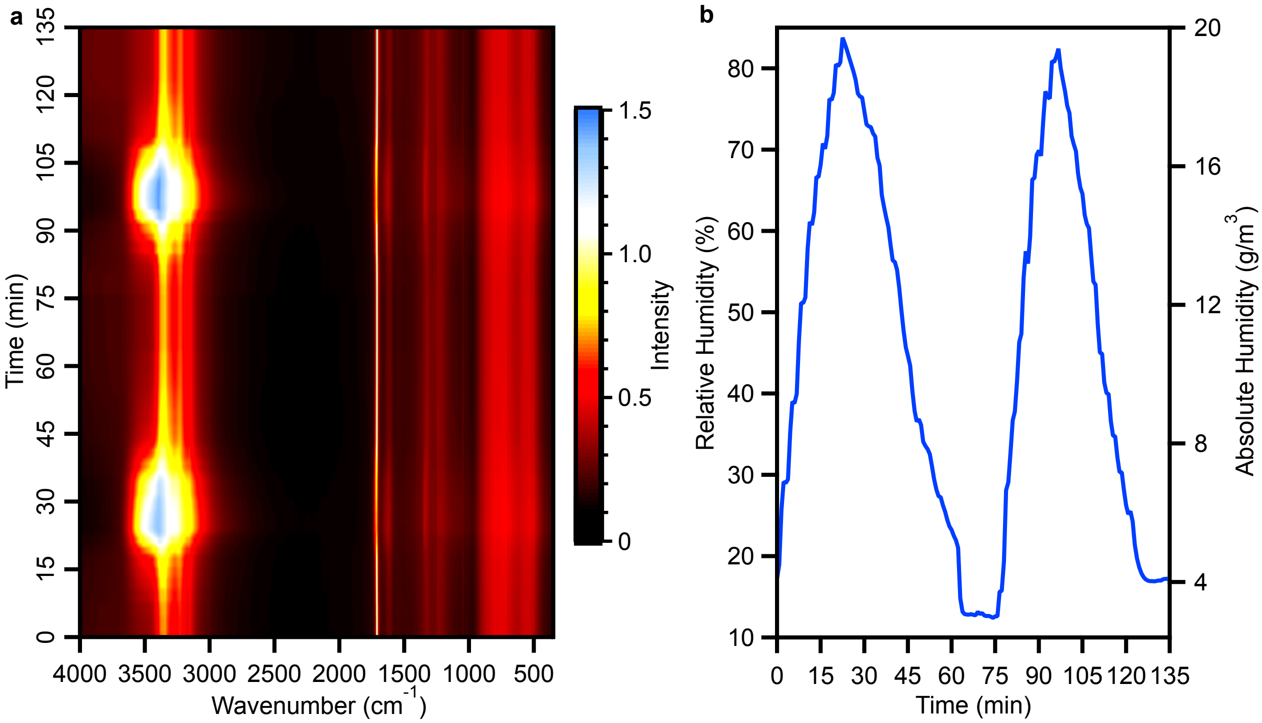

Supplementary Figure 13 | Effect of hygrochromic cycling on the molecular interactions. a Diffuse-reflectance infrared Fourier-transform spectroscopy (DRIFTS) of composite $FA_{n+1}Pb_nI_{3n+1}$ films as the relative humidity (RH) was varied as a function of time according to **b**. RH was increased at 3.2 ± 0.2 % min^{-1} and decreased at 1.9 ± 0.3 % min^{-1}. The temperature was maintained at 24.8 ± 0.4 °C and the atmospheric pressure in Golden, CO was 752 mmHg.

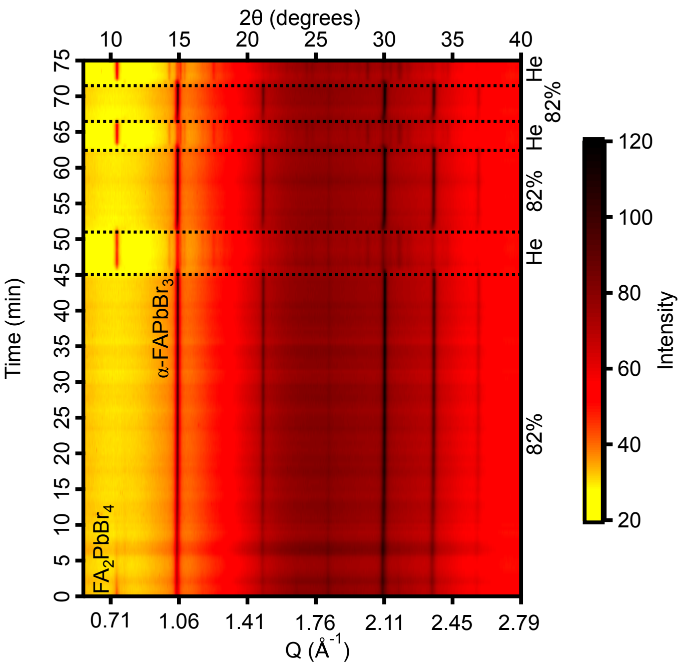

Supplementary Figure 14 | Structural evolution and reversibility during chromic cycling. *In-situ* wide angle X-ray scattering (WAXS) data collected on a $FA_{n+1}Pb_nBr_{3n+1}$ film over three cycles of alternating 82% relative humidity (RH) and He flow.

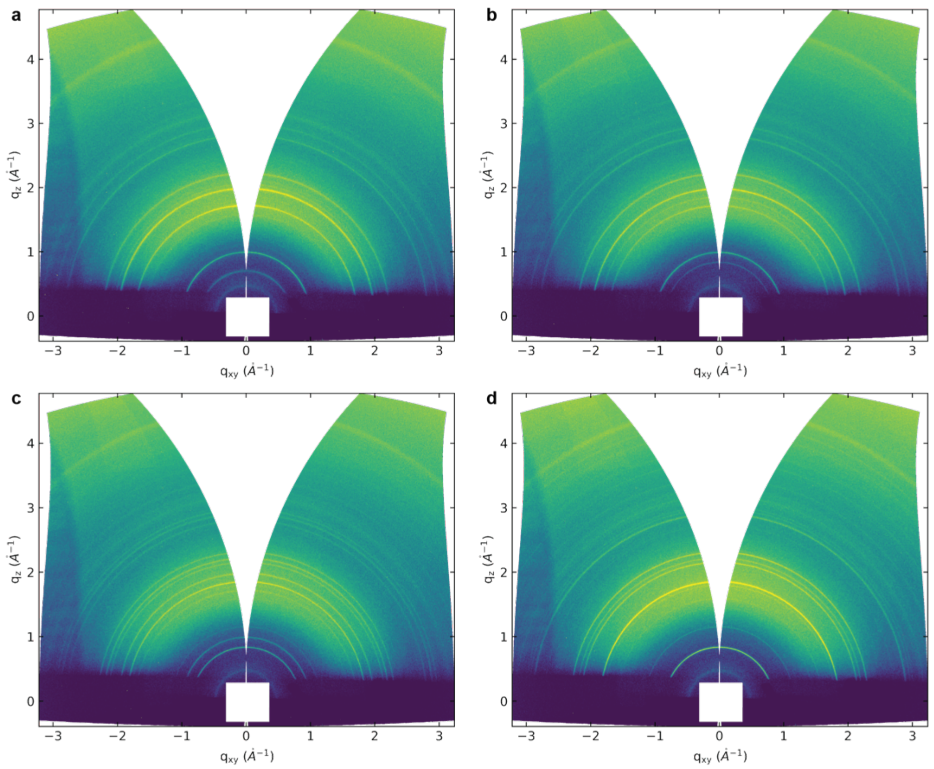

Supplementary Figure 15 | Effect of cycling on texturing. Representative 2D wide angle X-ray scattering (WAXS) images of a composite $FA_{n+1}Pb_nI_{3n+1}$ film exposed to 82% relative humidity (RH) air flow for 0 min **a**, 10 min **b**, 15 min **c**, and 30 min **d**. These images show polycrystalline films without texturing induced over time.

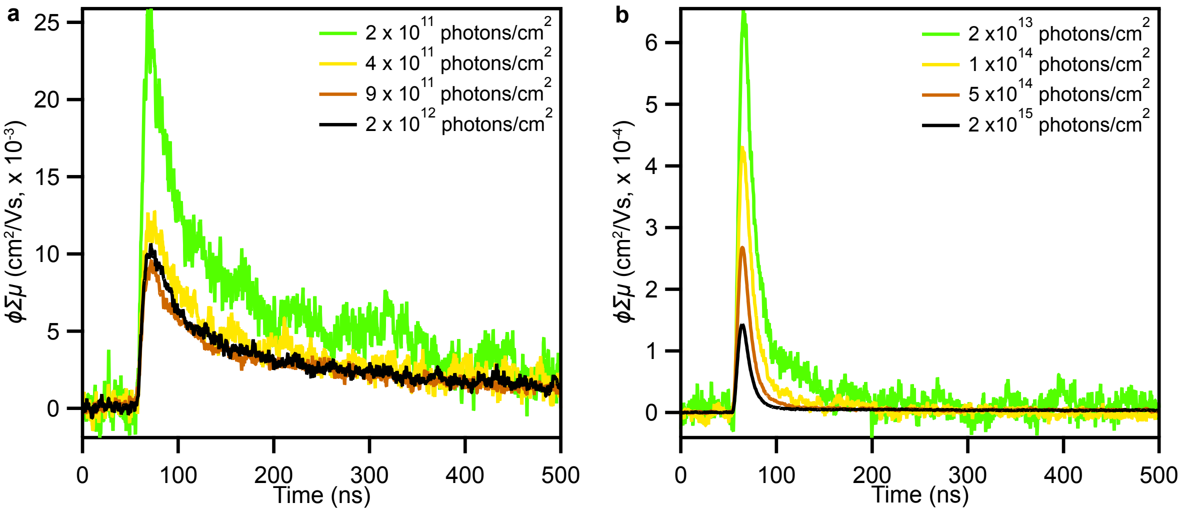

Supplementary Figure 16 | Evolution of $\phi\Sigma\mu$ values in hygrochromic $FA_{n+1}Pb_nI_{3n+1}$ films. Representative flash-photolysis time-resolved microwave conductivity (fp-TRMC) transients collected on a $FA_{n+1}Pb_nI_{3n+1}$ film at **a** 78% relative humidity (RH), brown phase and **b** 20% RH, yellow phase.

Supplementary Table 1. Summary of flash-photolysis time-resolved microwave conductivity (fp-TRMC) data for all yellow, orange, and brown $FA_{n+1}Pb_nI_{3n+1}$ films used for averaging. <t> is the amplitude-weighted average time constant obtained from biexponential fits from selected transients.

**Sample #**	**Color**	**RH (%)**	**Average $\phi\Sigma\mu$ ($cm^2\ V^{-1}\ s^{-1}$)**	**Average <t> (ns)**
1	Yellow	20 ± 3	0.006 ± 0.001	11 ± 3
2	Yellow	20 ± 3	0.003 ± 0.001	
3	Yellow	20 ± 3	0.0016 ± 0.0003	36 ± 9
1	Orange	70 ± 3	0.015 ± 0.009	27 ± 6
2	Orange	70 ± 3	0.012 ± 0.001	22 ± 5
3	Orange	70 ± 3	0.013 ± 0.006	20 ± 6
1	Brown	78 ± 3	0.010 ± 0.001	20 ± 3
2	Brown	75 ± 3	0.02 ± 0.01	51 ± 11
3	Brown	78 ± 3	0.025 ± 0.003	90 ± 6

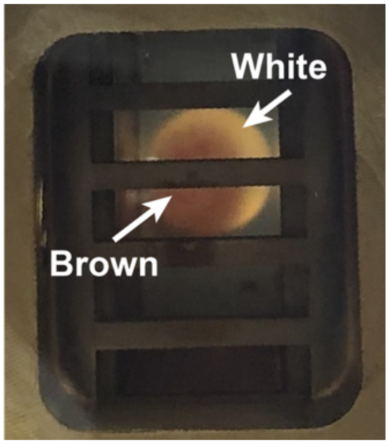

Supplementary Figure 17 | Transformation of white to brown color during flash-photolysis time-resolved microwave conductivity (fp-TRMC) acquisition. Image of the fp-TRMC holder showing that a $FA_{n+1}Pb_nI_{3n+1}$ film subjected to 82% relative humidity (RH) turns brown upon exposure to the various fp-TRMC fluences. Slight coloring of white phase is caused by the sample holder.

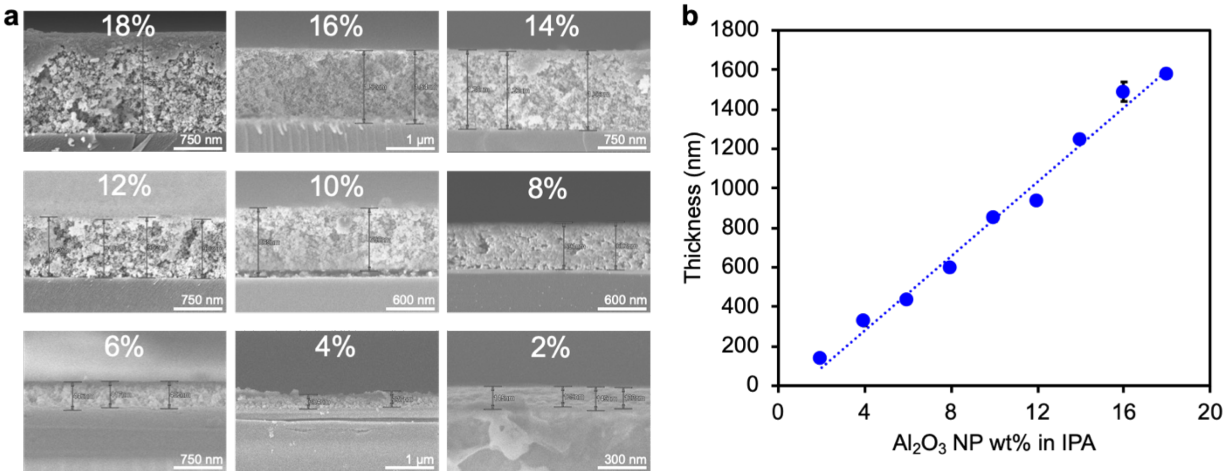

Supplementary Figure 18 | Control of scaffold thickness. **a** Representative cross-sectional scanning electron microscopy (SEM) images and **b** thickness variation of Al_2O_3 nanoparticle (NP) scaffolds prepared with precursors of varying wt% of Al_2O_3 NP's in isopropyl alcohol (IPA). Note that error bars are included in **b** for all data points.

Supplementary Figure 2. RH conversion table.

**Table 2.** Relative vs. Absolute Humidities	
RH (%)	AH (g m^{-3})
20	4.61
40	9.23
60	13.84
65	15.00
70	16.15
75	17.31
78	18.00
80	18.46
82	18.92

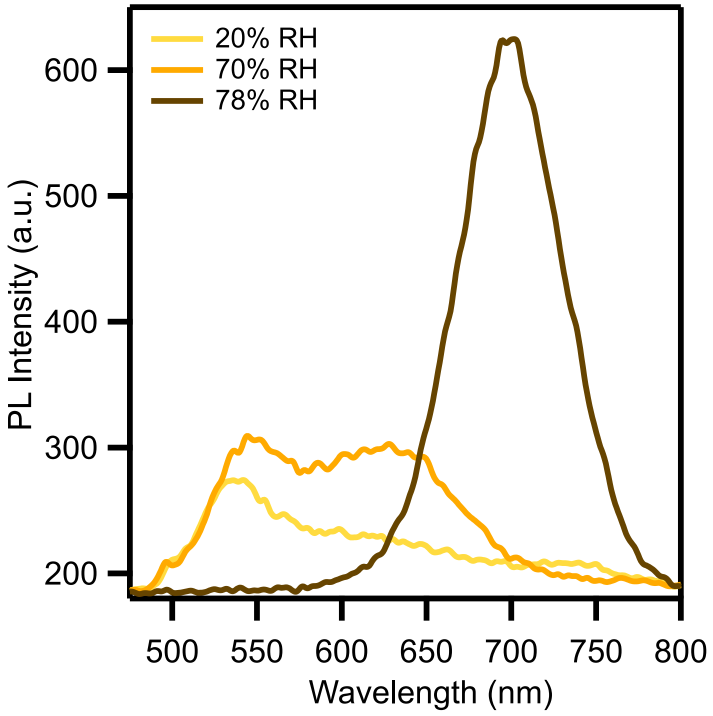

Supplementary Figure 19 | Photoluminescence (PL) collected during flash-photolysis time-resolved microwave conductivity (fp-TRMC) measurements. Representative uncorrected PL data of composite $FA_{n+1}Pb_nI_{3n+1}$ films exposed to varying relative humidity (RH) during fp-TRMC measurements. A 500 nm long pass filter was used to exclude scattered excitation light.

Supplementary References

1. Gratia, P. *et al.* The many faces of mixed ion perovskites: unraveling and understanding the crystallization process. *ACS Energy Lett.* **2,** 2686-2693 (2017).
